# Potential Occupational Exposures and Health Risks Associated with Biomass-Based Power Generation

**DOI:** 10.3390/ijerph120708542

**Published:** 2015-07-22

**Authors:** Annette C. Rohr, Sharan L. Campleman, Christopher M. Long, Michael K. Peterson, Susan Weatherstone, Will Quick, Ari Lewis

**Affiliations:** 1 Electric Power Research Institute, Palo Alto, CA 94304, USA; 2 American College of Medical Toxicology, Phoenix, AZ 85028, USA; E-Mail: campleman.toxic@gmail.com; 3 Gradient, Cambridge, MA 02138 USA; E-Mails: clong@gradientcorp.com (C.M.L.); mpeterson@gradientcorp.com (M.K.P.); alewis@gradientcorp.com (A.L.); 4 E. ON Technologies (Ratcliffe) Ltd., Ratcliffe on Soar, Nottinghamshire, NG11 0EE, UK; E-Mails: susan.weatherstone@eon.com (S.W.); will.quick@eon.com (W.Q.)

**Keywords:** biomass, occupational health, bioaerosols, particles, combustion

## Abstract

Biomass is increasingly being used for power generation; however, assessment of potential occupational health and safety (OH&S) concerns related to usage of biomass fuels in combustion-based generation remains limited. We reviewed the available literature on known and potential OH&S issues associated with biomass-based fuel usage for electricity generation at the utility scale. We considered three potential exposure scenarios—pre-combustion exposure to material associated with the fuel, exposure to combustion products, and post-combustion exposure to ash and residues. Testing of dust, fungal and bacterial levels at two power stations was also undertaken. Results indicated that dust concentrations within biomass plants can be extremely variable, with peak levels in some areas exceeding occupational exposure limits for wood dust and general inhalable dust. Fungal spore types, identified as common environmental species, were higher than in outdoor air. Our review suggests that pre-combustion risks, including bioaerosols and biogenic organics, should be considered further. Combustion and post-combustion risks appear similar to current fossil-based combustion. In light of limited available information, additional studies at power plants utilizing a variety of technologies and biomass fuels are recommended.

## 1. Introduction

Biomass-fueled power generation will contribute to reaching international targets for renewable production of electricity and related greenhouse gas emissions reductions through new construction or re-powering of existing coal-fired units [1]. Biomass combustors, common in small scale, industrial boiler, or cogeneration (heat/power) applications, have now been developed for electricity generation at a larger utility scale (over 50 megawatts (MW) thermal input) [2]. As with other solid fuel power plants, facilities using biomass as the primary combustion source can provide a reliable source for base load, cycling, and on-demand situations. However, as with any emerging or scaled-up technology, evaluation of environmental and occupational health impacts requires an understanding of the properties and characteristics of the fuel, as well as consideration of plant design, fuel processing, handling and storage [3].

In the case of occupational health and safety (OH&S), biomass combustion may result in several unique worker exposures relative to petroleum or coal-based fuels. These differences may be due both to the combustion process itself and the introduction of new occupational tasks related to biomass handling, storage and processing. Though extensive data from utility-scale operations are limited, occupational information can be gleaned from small-scale biomass technologies or related industries, such as waste handling and forestry [4,5]. This review focuses on the potential for occupational exposure and related health risks specific to biomass-based electricity generation, primarily for direct-fired, stand-alone technologies. It should, however, be noted that other biomass energy conversion processes, such as co-firing with coal, gasification, pyrolysis and anaerobic digestion have similar OH&S issues around biomass handling and, where available, experiences from these systems have been drawn upon. This review does not discuss the potential for health effects at the population level due to ambient emissions, or residential in-home exposures due to wood or other biomass burning (see [6] for a good review of this topic).

For the most part, it is possible to separate processes at power plants into three groups: pre-combustion (handling, storage, fuel preparation), combustion (including flue gas treatment), and post-combustion (ash and by-product handling). Each of these groups has its own inherent OH&S issues and hence this review follows a similar categorization. Following a discussion of the literature, testing results for dust, fungal and bacterial levels at two power stations are presented.

## 2. Summary of Available Technologies and Fuel Types

Combustion technologies used (or proposed) for modern biomass-fueled, direct-fired power plants vary by design, fuel flexibility, and environmental considerations. As such, the degree and type of emissions control technologies required to meet any required emission limits for pollutants of regulatory concern also influence OH&S issues. Biomass varies substantially in composition and fuel characteristics, so some combustion technologies may be more suitable than others for a particular biomass feedstock, depending on factors such as availability, composition and moisture content. The combination of fuel and boiler type chosen establishes the relative combustion efficiency, temperature range, and other combustion characteristics that influence the quantities, types and chemical composition of the solid waste to be handled post-combustion (ash and air pollution control residues). These factors, along with influences such as local pollution control regulations, also govern the choice of control technologies and ultimately the relative risks associated with worker exposure to potentially hazardous substances from combustion and post-combustion handling processes [2,3,7,8]. In direct-fired, 100% biomass combustion for power generation, combustion within a given boiler produces high-pressure steam for driving a turbine [9]. Table 1 provides a summary of the major types of stand-alone, direct-fired biomass technologies; the two most common combustion boiler types for dedicated biomass combustion are generally of a stoker (grate) or fluidized bed design. Table 2 provides a summary of available emission control technologies and related environmental exposures of potential concern for these two common designs; it should be noted, however, that not all technologies can be used with all biomass fuels.

In addition to these stand-alone technologies, a number of large (up to 660 MWe) pulverized coal units in Europe have recently been converted to combust 100% biomass, although this type of boiler is not generally considered the most suitable for a new build biomass plant due to the high level of biomass pre-processing required (such as drying and pelletizing).

A wide variety of biomass fuels are in current use for electricity generation. These include agricultural residues, such as straw, olive cake, and palm kernels, wood chip and wood residues, and specially grown “energy crops” such as miscanthus and switchgrass. The choice of the fuel (or mixture of fuels) used in a particular boiler depends on a number of factors, including availability of sufficient quantities (taking into account seasonality), fuel quality, potential negative impacts on the boiler, and price. In some countries, the definition of biomass also includes waste materials such as sewage sludge and post-consumer wood (including panel products such as particleboard). Levels of contaminants such as heavy metals can be significantly higher in these waste materials than for “clean” biomass types (e.g., see the Phyllis2 database [10]. As a result, their use is often subject to tighter regulatory controls. For example, the EU’s Industrial Emissions Directive includes emission limits for biomass combustion in the same section as fossil fuels, but plants using demolition wood must meet the stricter waste incineration limits [11].

**Table 1:** Summary of available large-scale, standalone biomass combustion technologies for electricity generation.

Direct Fired Technology	Common Fuel Types	Biomass Feed Size (cm)	Moisture Content (%)	Generation Capacity (MW)
Pile burners	Wood or agricultural residues (excl. wood flour)	Limited by grate size and feed opening	<65	4 to 110
- with underfire stoker	Sawdust, select bark (“non-stringy”), shavings, chips, “hog” fuel	0.6–5	10–30	4 to 110
Stoker grate boilers	Sawdust, select bark (“non-stringy”), shavings, end cuts, chips, “hog” fuel, sander dust	0.6–5	10–50	4 to 300
Suspension boilers				
- Cyclonic	Sawdust, select bark (“non-stringy”), shavings, wood flour, sander dust	<0.6	<15	<30
- Air spreader-stoker	Wood flour, sander dust, processed sawdust, shavings	0.1–0.15	<20	1.5 to 30
Fluidized-bed combustor	Low alkali fuels: wood residues or peat	<5	<60	Up to 300
- with underfire stoker	Sawdust, select bark (“non-stringy”), shavings, chips, “hog” fuel	0.6–5	10–30	4 to 110
- with underfire stoker	Sawdust, select bark (“non-stringy”), shavings, chips, “hog” fuel			

Summarized from [2] and [12].

**Table 2:** Substances of significance for health and corresponding emission control options for stoker or fluidized bed boilers.

Air Pollution Control or Environmental Target	Emission Control Options	
	Stoker Boiler	Fluidized Bed Boiler
Typical post-combustion air pollution control	PM—Cyclones, ESP, FF NOx—SNCR, SCR (only applicable for low alkali fuels) CO—oxidation catalysis SOx/HCl—IDSIS, SDA, DS (with FF), FGDw	PM—ESP and FF NOx—SNCR, SCR (only applicable for low alkali fuels) CO—generally absent SOx/HCl—In furnace injection, IDSIS, SDA, DS, FGDd (with FF)
Low sulfur oxide (SOx) combustion	Not possible (in furnace)	Some reduction possible through limestone addition to bed material
Low NOx combustion	Air staging	Generally low inherent NOx (due to lower temperature), air staging, flue gas recirculation
Low CO formation	Difficult (lower combustion efficiency)	Generally low due to higher combustion efficiency

Summarized from: [2,7,8,13]; CO = Carbon Monoxide; DS = Dry sorbent; ESP = Electrostatic Precipitator; FF = Fabric Filter or Baghouse; FGDd = Dry Flue Gas Desulfurization; FGDw = Wet Flue Gas Desulfurization; HCl = Hydrogen Chloride; IDSIS = In Duct Sorbent Injection System; NOx = Nitrogen Oxides; PM = Particulate Matter; SCR = Selective Catalytic Reduction; SDA = Spray Dryer Absorber; SNCR = Selective Non-Catalytic Reduction; SOx = Sulfur dioxide.

## 3. Potential Occupational Exposures

Evaluating potential occupational exposures at biomass-fueled power generation facilities is complicated not only by the wide variety (and mixtures) of fuel types, but also by the variety of facility designs and lack of detailed exposure monitoring data reported in the literature. The focus of this section is on exposures associated with fuels used at these facilities (pre-combustion, stack emissions, and post-combustion), as opposed to other secondary occupational exposures (*i.e.*, forklift/truck traffic, diesel generators, *etc.*). Evaluation of a biomass feedstock generally includes analyses for energy content, fuel properties (including moisture and ash content), and major fuel elements (carbon, hydrogen, nitrogen, sulfur, chlorine) [14], as well as more minor components capable of influencing plant operations, including the main mineral components of the ash and levels of heavy metals [15,16]. These physiochemical properties also influence the type of emissions (air, water and solids), environmental impacts, and plant control requirements. Just as the availability and type of the source fuel(s) influences the ability to design, site, and operate a large-scale biomass combustion plant [17], it also determines the nature of the operational waste streams and the associated potential for worker exposure. As with other combustion-based power plants, biomass-fueled facilities produces emissions to air and water, as well as solid byproducts such as ash and pollution control residues.

Due to the limited data regarding occupational biomass exposures in the power generation sector, potential worker exposures—particularly those unique to biomass *versus* other fuels—are described from similar occupational exposures as needed, such as wood pellet or other biomass waste management. Exposed populations of interest are identified, and relevant exposure sources and routes are discussed. The section also identifies substances of significance to health (SSHs) at these facilities, and further discuss SSHs that may have different exposure profiles than at traditional fossil fuel power generation facilities.

### 3.1. Overview of Exposure Sources and Routes

In general, three primary sources of exposure should be considered for an occupational risk assessment of a biomass-fueled generation facility: the biomass fuel itself (pre-combustion), biomass combustion emissions (usually associated with the boiler or stack), and exposure to the resulting ash residue (post-combustion). Some exposures may be common to multiple stages. For example, workers may be exposed to gaseous pollutants and particulate matter (PM) generated from biomass handling, transport, storage, and agitation, as well as from post-combustion ash. Numbers of workers and their typical tasks vary between installations, but a basic overview is provided in Table 3.

The inherent physiochemical characteristics, including the amount of cellulose, hemicelluloses, and volatile organics, in common biomass sources such as straw, wood pellets and chips, may be expected to influence pre-combustion exposures. As well as dust from the material itself, biomass may contain an inhalable bioaerosol component, comprised of microorganisms and endotoxins [18]; these materials may be released during industrial handling [19,20,21]. In general, exposure levels in wood handling industries differ substantially by the type and size of biomass, by temperature and humidity, and by the specific task (e.g., transport, shredding, agitation) [20]. Primary exposure routes are likely to be through inhalation of particulate, bioaerosols and volatile compounds and dermal contact, although there is also the risk of mechanical irritation of the eyes. Ingestion is a less likely route of exposure, although contamination of welfare areas with biomass may be an issue in some cases where controls are inadequate.

For onsite personnel, the primary exposure route is likely to be associated with the pre-combustion release of PM, bioaerosols, and volatile organics from the biomass during storage and handling operations. Although combustion of biomass produces pollutant gases and PM, exposure to these is considered to be a risk mainly at the very small scale (such as in domestic heating and cooking); at the utility scale, plant design and control should minimize the risk of worker exposure to combustion products.

For some plant workers, there is also the potential for exposure to post combustion products, particularly ash. Different combustion technologies produce ash with differing characteristics, which are further modified by emissions control systems. At the utility scale, it is usual for different ashes to be handled separately, with streams labeled as “bottom ash” and “fly ash” most commonplace. The bottom ash, removed from the bottom of the boiler, is primarily composed of relatively unreactive, high melting point materials such as aluminosilicates; with fluidized bed boilers there is also a contribution from the bed material (often sand), as well as any limestone used for acid gas control. In contrast, the fly ash consists of those inorganic components that have volatilized in the furnace before condensing as the gas cools, as well as fine non-volatile ash that has become entrained in the flue gas before being collected in control devices such as filters and electrostatic precipitators (ESPs). Many of the volatile trace elements contained in the fuel are concentrated in this fly ash. Where dry sorbents are added to the flue gas for pollutant control (e.g., lime for acid gas abatement or activated carbon for heavy metal control), these are also removed with the fly ash. Some plants may have multiple ash capture stages to reduce the proportion of the ash contaminated with air pollution control sorbents.

Although it may be assumed that the highest risk of exposure to ash is among those personnel involved in its handling and storage, there are other groups of workers who may also be at risk. Where ash handling systems are not fully enclosed, airborne ash releases may affect all personnel, while those workers working on repair and maintenance within the boiler are likely to be exposed to ash in the form of furnace deposits. It should be noted that these boiler deposits could have different chemical characteristics to the bulk ash; for example, they could be enriched in those metals that preferentially condense into the deposit at particular furnace temperatures [22]. While potential exposure to ash should be limited by process controls (such as enclosure of handling systems) wherever possible, with Personal Protective Equipment (PPE) used by workers to reduce any residual risk, the efficiency of these controls can vary widely. The effectiveness of PPE in particular is heavily influenced by factors including training, proper fit (a particular issue with respiratory protection), safety culture, management enforcement, and workers’ own perception of risk. Exposure routes of interest for ash include inhalation and dermal contact during transfer and transport processes, with incidental ingestion of ash or dust comparatively less important. Biomass ash can also be highly alkaline, presenting a risk of irritation and corrosiveness due to pH alone, particularly in contact with skin and eyes.

### 3.2. Substances of Significance to Health

#### 3.2.1. Pre-Combustion Exposures

Pre-combustion exposure to biomass materials is influenced by the unique physiochemical properties of the fuel. A limited number of European studies have reported ambient PM concentrations within facilities associated with biomass combustion, processing or handling; these have often focused on bioaerosols, including bacteria, fungi, endotoxin, and other related markers [4,5,23,24,25]. There has also been significant interest in exposure to gaseous species, mainly carbon monoxide (following a number of fatal incidents during transport and storage of wood pellets), but also volatile organics [26,27,28,29,30]. In general, these studies have focused on area monitoring or overall personnel exposure assessment, with minimal or no worker task specification. Table 4 summarizes the SSHs identified from the literature for biomass handling, processing (e.g., wood pellets), and storage at either biomass power facilities or related industries such as wood pellet production.

#### 3.2.2. Combustion-Related Exposures

The major combustion SSHs emitted from biomass-fueled power generation facilities are similar to those from traditional fossil fuel generation facilities. Concentrations of these substances in the flue gas can be influenced by factors such as fuel chemical composition, boiler design, pollutant control systems, and combustion conditions, and so can vary considerably between different facilities. In addition to criteria pollutants such as PM, carbon monoxide (CO), sulfur oxides (SOx), and nitrogen oxides (NOx), a number of different volatile organic compounds (acrolein, aldehydes) and some associated persistent semi-volatile compounds (PAHs, dioxins/furans) may also be present, although data regarding their presence—and more especially their concentrations—in the flue gas are often limited. Emitted PM also contains mineral and metal species. The US EPA AP-42 guidance does provide emission factors for a large number of organic species from wood residue combustion in boilers; these factors were last updated in 2001 [31]. The boilers which have provided the data used to generate these factors are generally industrial-scale rather than utility-scale units, being primarily used to utilize residues from wood processing facilities and pulp mills. In many cases, emission factors presented are derived from only one or two measurements. Where there are multiple measurements available, the range of values often spans several orders of magnitude. As a result, these factors may not reflect current practice at the utility scale, particularly in terms of boiler design (most tests were undertaken on stoker or Dutch Oven-type boilers) and flue gas clean up. Non-woody biomass fuels are not considered in AP-42, with the exception of a limited amount of data provided for bagasse use in sugar mills. It should be noted that as the size of the installation increases, there is greater scope for optimization of the combustion system, improving efficiency and reducing air emissions associated with poor combustion. In many countries, there are also legally mandated emission limits on major pollutants to air for plants over a specified thermal input, and these limits often decrease as plant size increases (see for example [11]). The controls required to meet the limits for major pollutants often also provide a co-benefit removal of minor species (for example, systems for sulfur oxide reduction can also reduce other acidic gases) [13]. Emission rates (per unit of output) for large-scale generation plants can therefore be significantly lower than for smaller industrial units. Table 5 provides a summary of the types of substances that may be of interest to occupational health in this industry; however, it should be noted that many of these substances are associated with combustion processes in general, not biomass combustion specifically.

Although few quantitative data on SSHs at biomass combustion facilities exist, a number of studies provide information on the relative stack emissions at biomass facilities compared to traditional fossil-fueled facilities. For example, biomass fuels generally have lower levels of mercury and sulfur than coal, and thus mass emissions of SOx and mercury from these facilities would likely be lower (assuming similar control technologies) [32]. Chlorine levels in biomass are more variable, but for wood (the most common biomass fuel used for large-scale generation) chlorine content is typically very low, which may lead to low emissions of chlorinated dioxins and furans. However, on the other hand, the heterogeneous nature of biofuels (as compared to coal) might lead to less efficient combustion and possibly the formation of proportionately more of these chemicals for the same chlorine content in the two fuels [32,33,34]. Emissions of PM and NOx depends on the levels of ash and nitrogen in the fuel, the combustion system, and the emissions control technologies used. At the utility scale it could be expected that PM and NOx emissions would be lower than for coal combustion (except for those coal plants fitted with selective catalytic reduction systems for NO_x_), but higher than for light oil or natural gas combustion [5,36].

While the types of SSHs emitted are fairly well understood, there are very few data on concentrations that might be relevant to assessing occupational risks *versus* concentrations related to ambient releases. The design and operation of modern biofuel plant is such that the probability of release of flue gas into the plant itself should be low, and therefore assessment of risk of worker exposure to combustion products based on composition of stack emissions is likely to overestimate risk.

#### 3.2.3. Post-Combustion Related Exposures

The majority of inorganic material associated with the biomass fuel is recovered from the boiler as ash. As the composition of mineral matter in different biomass fuel varies, so does the ash, with additional variability introduced by the use of non-fuel materials, such as sand or other minerals, as the bed material in fluidized bed boilers, along with the use of sorbents for flue gas treatment. In large-scale boilers, multiple ash streams are often produced with different chemical properties. For example, in fluidized bed boilers the bottom, or bed, ash consists of a mixture of fuel ash, bed material, and coarse contaminants of biomass (such as stones). Certain volatile elements, including sulfur, chlorine, alkali metals, and some heavy metals, are depleted in the bottom ash, as the temperatures in the boiler are sufficient to vaporize them and they exit the boiler in the flue gas. In contrast, the fly ash (or filter ash) consists of material fine enough to be carried by the flue gas and can be enriched in the volatile elements as they condense out onto the ash as the flue gas cools.

**Table 3:** Typical power plant tasks and exposures.

Job Type	Tasks	Potential Exposures
Trucker	Transport of biomass to site (road/rail) Loading and discharge of material Transport of ash	Biomass dust and bioaerosols generated during biomass loading and discharge Ash dust generated during loading and discharge Diesel exhaust from vehicles
Fuel Handling Plant operative	Transport of biomass through the site Storage of biomass Fuel preparation (milling *etc.*)	Biomass dust and bioaerosols generated during biomass handling and milling Off-gases from storage Direct contact with moldy biomass
Cleaner	Removal of dust deposits from plant	Generation of airborne biomass dust, bioaerosols and ash through disturbance of deposits Potential for direct contact with moldy biomass
Maintenance engineer	Maintenance of plant equipment during normal operation	Generation of airborne biomass dust, bioaerosols and ash through disturbance of deposits Potential for exposure to combustion gases
Outage contractor	Repair of plant items during shutdown periods (particularly within the boiler)	Generation of airborne biomass dust, bioaerosols and ash through disturbance of deposits Direct contact with ash deposits within the boiler (often confined spaces)
Ash handling plant operative	Removal of ash from the boiler Transport to storage	Direct contact with ash
Other plant personnel	Various	Fugitive dusts from fuel and ash handling plants Combustion gases

**Table 4:** Identified substances of significance to health (SSHs): pre-combustion.

SSH Class	COI	Source	Industry	Reference(s)
Particulate Matter	Wood dust	Raw or processed material Straw, wood chips, pellets	Forestry Wood pellet production Biomass generation Biomass laboratory	[25,37,38,39,40]
Bioaerosols	Microbial (Fungi/Bacteria)	Component of PM Wood chips or pellets	Biomass power generation Fuel processing and handling	[23,40,41,42,43]
	Endotoxin	Component of PM Straw, grain, hay, organic waste	Biomass power generation	[21]
Volatile Organics (VOCs)	Aldehydes Total VOCs	Off gassing from sawdust Auto-oxidation of unsaturated fatty acids	Wood pellet production	[5,26]
Organics	Monoterpenes Resin acids	Components of PM, off gassing from sawdust	Wood pellet production Forestry, milling	[5,26,44]
Inorganic Gases	Carbon monoxide	Off gassing from raw materials	Wood pellet production, transport, storage	[28,29,30]

**Table 5:** Identified substances of significance for health (SSHs) and potential health effects: combustion and post-combustion.

SSH Class	SSH	Source	Refs	Health Effects Associated with Exposure Route		Refs
				Inhalation	Dermal/Eye	
Inorganic Gases	Carbon monoxide	Combustion	[45]	CNS; Miscarriage; Carboxylhemoglobinemia		[45,46]
	Nitrogen oxides	Combustion	[45]	URT and LRT	Irritation (Skin and Eye)	[45,48]
	Sulfur oxides	Combustion	[46]	Pulmonary function; LRT		[45,49]
	Acid aerosols (e.g., H_2_SO_4_)	Combustion	[47]	Pulmonary function	Irritation (Skin and Eye)	[45,49]
Hydrocarbons	1,3-Butadiene	Combustion	[45]	CNS; Stomach, Respiratory and Hematolymphopoietic Cancers		[45,50]
	n-Hexane	Combustion	[45]	CNS; Peripheral Neuropathy	Irritation (Eye)	[45]
	PAHs **^a^**	Combustion, Ash	[45,48,49]	Lung Cancer	Skin Cancer *	[51]
	Benzene	Combustion	[45]	Leukemia; Anemia; CNS		[45,52]
	Styrene	Combustion	[45]	CNS		[45]
Oxygenated organics	Acrolein	Combustion	[45]	URT; Pulmonary edema; Pulmonary emphysema	Irritation (Skin and Eye)	[45]
	Formaldehyde	Combustion	[45]	URT; Nose Cancer *	Irritation (Skin and Eye)	[45,53]
	Methanol	Combustion	[45]	CNS; URT	Eye Damage	[45,54]
	Acetic acid	Combustion	[45]	URT; Pulmonary function	Irritation (Eye)	[45]
	Catechol	Combustion	[45]	URT	Dermatitis; Irritation (Eye)	[45]
	Cresol (methylphenols)	Combustion	[45]	URT; Kidney; Liver	Skin Damage	[45,55]
	Hydroquinone	Combustion	[45]	CNS	Irritation (Eye)	[45,56]
	Fluorenone	Combustion	[45]	URT	Irritation (Eye)	[57]
	Anthraquinone	Combustion	[45]	Respiratory	Irritation (Skin and Eye)	[58]
Chlorinated organics **^b^**	Methylene chloride	Combustion	[45]	CNS; Peripheral Neuropathy; Liver and Lung Cancer *	Irritation (Skin and Eye)	[59,60]
	Methyl chloride	Combustion	[45]	CNS; Liver; Kidney; CNS *; Testicular *; Teratogenic *		[45,61]
	Dioxins/furans	Combustion	[45,48]	URT; Chloracne; Liver; Glucose metabolism	Chloracne	[62,63]
Particulate matter (PM)	PM_10_	Combustion/Condensation	[45]	Pulmonary function; URT	Irritation (Eye)	[64]
	PM_2.5_	Combustion/Condensation	[45]	Pulmonary function; URT	Irritation (Eye)	[22]
Inorganics	Aluminum (Al) **^c^**	Combustion	[45]	Pneumoconiosis; LRT		[45,66]
	Arsenic (As) **^e^**	Ash	[48,49]	URT and LRT; Lung Cancer		[45,67]
	Beryllium (Be) **^d^**	Ash	[48]	Beryllium disease;	Irritation (Skin)	[45,68,69]
	Cobalt (Co) **^d,e^**	Ash	[48]	Pulmonary function; Myocardial effects		[45,70]
	Magnesium (Mg) **^d^**	Combustion	[45]	URT; Pulmonary function; Metal fume fever	Irritation (Eye)	[71]
	Iron (Fe) **^d^**	Combustion	[45,49]	Pneumoconiosis; URT	Irritation (Skin and Eye)	[45,72]
	Manganese (Mn) **^f^**	Combustion	[45]	Neurobehavioral		[73,74]
	Zinc (Zn) **^h^**	Combustion	[45,49]	Metal fume fever; LRT and URT	Irritation (Skin and Eye)	[45,75,76]
	Nickel (Ni) **^d^**	Combustion, Ash	[45,48,49]	Pneumoconiosis; Nasal and Lung Cancer	Dermatitis	[45,77,78]
	Copper (Cu) **^d^**	Combustion	[45,49]	URT; Metal fume fever	Irritation (Eye)	[45,79]
	Lead (Pb) **^f,g,h,i^**	Combustion	[45,49]	CNS and PNS; Hematologic; Nephropathy		[45,80]
	Mercury (Hg) **^d,f^**	Ash	[48]	CNS and PNS; Kidney		[45,81]
	Chromium (Cr) **^d^**	Combustion, Ash	[45,48,49]	Pulmonary function; Lung Cancer	Irritation (Skin)	[45,82]
	Cadmium (Cd) ^d,i^	Combustion	[45]	Pulmonary function; Kidney		[45,83,84]
	Quartz	Ash	[48]	Pulmonary fibrosis; Chronic silicosis; Lung cancer *		[45,85]

CNS—central nervous system; LRT—lower respiratory tract; PNS—peripheral nervous system; URT—upper respiratory tract; * Endpoints derived from animal studies; **^a^** Oral exposure—Animal bioassays positive for reproductive/developmental effects and stomach cancer; **^b^** Assumes chlorine in fuel; **^c^** Oral exposure—Animal bioassay positive neurotoxicity; **^d^** Oral exposure—Human gastrointestinal toxic effects observed for Be, Co, Mg, Fe, Ni, Cu, Hg, Cr, Cd; **^e^** Oral exposure—Human skin toxicity observed for As (and cancer) and Co; **^f^** Oral exposure—Human CNS effects observed for Mn and Pb; **^g^** Oral exposure—Human PNS effects observed for Pb; **^h^** Oral exposure—Human hematologic effects observed for Zn and Pb; **^i^** Oral exposure—Human kidney toxicity observed for Pb and Cd.

Two studies provide information on levels of SSHs in biomass boiler room dust (likely to consist of a mixture of pre-combustion and post-combustion material), and others have reported qualitative aspects of exposure. Cohn *et al.* [23] reported levels of PAHs and selected trace metals in three dust samples collected from the boiler room at a straw-burning biomass generation facility in Denmark (Table 6). Madsen and Sharma [18] performed an analysis on a single sample of dust collected in the boiler room of a straw-fueled biomass plant and found that the primary inorganic elements present were potassium, calcium, and sodium. Other elements included aluminum, magnesium, iron, manganese, phosphorus, zinc, nickel, copper, lead, chromium, and cadmium (Table 6). Although different analytes were targeted in each study, the overlapping analyzed components were roughly similar with respect to concentration, with the exception of nickel, which was higher in the Madsen and Sharma study than the Cohn *et al.* study. It should be noted that the number of samples was extremely low in both studies, limiting interpretability of the findings.

**Table 6:** SSHs in biomass power generation boiler room dust.

SSH	Madsen *et al.* [18] *N* = 1 Concentration (ppm)	Cohn *et al.* [23] *N* = 3 Concentration Range (ppm)
K	303,154	−
Ca	53,061	−
Na	44,266	−
Al	6789	−
Mg	5892	−
Fe	16,434	8100–28,000
Mn	361	−
P	1890	−
Zn	1770	1050–15,700
Ni	568	30–125
Cu	530	300–525
Pb	127	115–150
Cr	38	20–50
Cd	5	−
Li	−	4.8–15
As	−	5–15
PAH	−	145–880

− not analyzed.

## 4. Potential Occupational Risks

The potential occupational health impacts of biomass combustion in power generation remain poorly defined, and as a result, there is limited guidance available to inform monitoring and health surveillance best practice guidance. The following section classifies potential occupational risks related to biomass into pre-combustion, combustion, and post-combustion categories. Because of the limited availability of sector-specific studies, information from related industries, uncontrolled combustion, or ambient-focused studies is utilized. Unfortunately, these studies cannot be relied upon to provide specific information related to occupational scenarios using controlled generation technologies, but can serve as a guide for future worker health and safety research.

### 4.1. Pre-Combustion Risks

In combination with the sparse information regarding exposures of biomass-based generation workers, a lack of epidemiologic studies limits the ability to establish potential associations or speculate on the role of biomass in any potential adverse health effects in workers. However, ancillary data from related industries can help to define constituents of potential concern for future study.

#### 4.1.1. Bioaerosols

A number of case studies have associated occupational health effects with exposure to microorganisms in wood chip dust. Exposure to fungi from stored chipped wood used for heating has been linked to respiratory allergies and hypersensitivity pneumonitis [41,42,43,86]. In the wood-processing industry, dose-response relationships have been reported between endotoxin levels and respiratory symptoms [87], with significantly higher prevalence of respiratory symptoms such as cough and chronic bronchitis among woodworkers than in the control group. However, the microbial content of fuel biomass used in large-scale power generation has not been extensively reported, making it difficult to extrapolate to potential exposure levels of concern. Cases of extrinsic allergic alveolitis (EAA) have been identified in connection with the use of wood chips for heating. van Assendelft *et al.* [42] reported that EAA was associated with endotoxins for *Penicillium* in two farmers, the first of whom used green pine and alder chips, and the second who used birch, osier, and alder woods. Both cases reported respiratory symptoms and malaise after handling wood chips. Furthermore, in the second case levels of molds, including *Penicillium* and *Aspergillus*, were high on the surface of the wood chip, despite the storage area being cleaned and no visible signs of mold growth in the material. EEA was also diagnosed in the case of a maintenance worker in a sawmill which processed spruce and Douglas fir woods [88]. Immunological testing suggested sensitization of the worker to *Trichoderma konigii*, exposure to which was believed to be associated with the use of damp logs in the sawmill.

Ławniezek-Wałczyk *et al.* [89] reported the results of bioaerosol sampling at a coal-fired power plant that was also co-firing sunflower seed pellets and wood chips. Analysis of samples collected from nine plant locations plus an outdoor reference location with MAS (*N* = 4 per location) and Andersen six-stage (*N* = 20) impactors showed that both bacterial and fungal spore levels were significantly higher within the plant than the reference case (all *t*-test *p* values < 0.05). Levels of airborne bacterial spores varied from 5.1 × 10^2^ cfu/m^3^ to 2.0 × 10^4^ cfu/m^3^ while fungal spore levels varied between 2.2 × 10^2^ cfu/m^3^ and 2.3 × 10^4^ cfu/m^3^. Levels were highest in the areas around the conveyor system, particularly where the biomass was in free-fall, such as during conveyor loading and transfer. Species analysis showed that fungal types included *Aspergillus* species (including *A. fumigatus*), *Mucor* spp., *Penicillium* spp., *Rhizopus stolnifer*, and a number of yeasts. Gram-negative rods identified included *Citrobacter* spp., *Pseudomonas* spp. (including *P. aeruginosa*, which can cause severe lung and urinary tract infections), and *Rahnella aquatilis*, while various Gram-positive *Bacillus*, *Micrococcus* and *Staphylococcus* species and thermophilic and mesophilic actinomycetes were also identified. The Polish Ministry of Health [90] classifies nine of the species identified as a “group 2” infection risk. Analysis of fresh samples of the biomass types used at the plant showed a similar mix of genus types, although the number of species identified was smaller.

Madsen *et al.* [25] examined the levels of different microbial indicators, including bacteria, actinomycetes, fungi, lipopolysaccharide, endotoxin, and muramic acid for various biomass stock (straw, wood chips, wood pellets, and wood briquettes) handling on a small pilot scale (particulate generated via rotating drum). Both the microbial content and overall “dustiness” varied by fuel type, analytical method, and biochemical indicator. Overall, straw generated more respirable particles (both by number and mass), total bacteria, and endotoxin *versus* wood chips, pellets or briquettes. Not unexpectedly, moisture content influenced particle generation, with higher moisture decreasing overall particle release. However, wood chips generated as much or more respirable PM than straw during initial handling (e.g., early generation rate in rotating drum test). By comparison, wood pellets and briquettes (both processed biomass stock) generated the lowest amount of microbial components, potentially indicating that non-microbial particles may be a greater concern for this type of biofuel. The densification process usually requires heat, and sometimes steam, decreasing the inherent microbial content of the material, while the low moisture content of the product (<10% is typical; higher moisture levels cause pellets to swell and break up into dust and so are avoided) limits its suitability as a growth medium for opportunistic microorganisms. However, these fuels may be prone to break-up during transport and handling, particularly if this involves multiple stages as in the case in large-scale supply, potentially releasing fine dust.

A follow-up study measured fungi, bacteria, actinomycetes, endotoxin, and *n*-acetyl-beta-d-glucoaminidase at five Danish biomass-fueled plants (straw and/or wood chips) at different seasonal time points [20]. Both personal worker and stationary area monitors were utilized to determine inhalable bioaerosols for an approximately 5- to 7-h window in fuel areas (e.g., storage), non-fuel areas (e.g., offices), and outdoors (e.g., local background). In total, 32 personal exposure measurements and 108 area samples were taken across the five plants over four days of monitoring (two in spring, two in autumn). Personal levels were converted to a time-weighted average (TWA). In summary, the authors considered levels of endotoxin (median personal exposure 55 EU/m^3^), bacteria (4.8 × 10^5^ cells/m^3^) thermophilic actinomycetes (1.3 × 10^4^ cfu/m^3^), and fungi (2.1 × 10^5^ spores/m^3^) to be high at all five biomass-fueled plants. As with the laboratory tests, the highest levels of endotoxin exposure were associated with straw (although *Aspergillus fumigatus* levels were highest at the wood chip plant). Work related to the straw shredder produced levels up to 119,000 EU·m^−3^. For perspective, this is orders of magnitude higher than the levels reported by Zock *et al.* [91] to affect lung function in potato processing workers (53 EU·m^−3^). In these Danish plants, 34% of workers handling straw or wood chips had exposure levels above 150 EU·m^−3^, and the overall median personal exposure of 55 EU·m^−3^ was higher than that observed by Rongo *et al.* [92] in small-scale wood industries. Levels of bacteria and fungi were also high in this study. For example, in 81% of study workers, personal exposures to mesophilic fungi were higher than levels previously reported to be associated with eye, nose and respiratory irritation (>10^4^ colony forming units (cfu) per m^3^ ) [93]. These levels are higher than previously reported in the wood processing [87] and milling [4] industries.

In further work, levels of fungal and bacterial components in PM_1_ were analyzed in samples taken from 14 Danish biofuel plants principally utilizing straw [95]. *N*-acetyl-β-d-glucosaminidase and (1→3)-β-d-glucans, both associated with fungi, were found in all PM_1_ samples (*N* = 29) at higher concentrations than in total dust, while cultivatable fungal spores were present in 6 of the samples and thermophilic actinomycetes in 23. Some research suggests a relationship between (1→3)-β-d-glucan and airway inflammation [96]. Few occupational exposure limits exist for bioaerosols, although the Dutch Expert Committee on Occupational Safety has recommended a health-based limit for endotoxin of 90 EU/m^3^ [97]. Recommended reference values of 1.0 × 10^5^ cfu/m^3^ for bacteria and 5 × 10^4^ cfu/m^3^ for fungi in industrial settings where organic dusts are present have also been proposed [89]. Eduard [93] identified a lowest observed effect level (LOEL) for diverse fungal species of 10^5^ spores/m^3^ in non-sensitized populations; however, for asthmatic patients with pre-existing allergy to *Penicillium* sp. or *Alternaria alternate*, LOELs to the sensitizing agent of 1 × 10^4^ spores/m^3^ and 2 × 10^4^ spores/m^3^ respectively were identified for reduced airway conductance.

Wouters *et al.* [98] reported on results from personal monitoring of workers for exposure to dust, endotoxin, and (1→3)-β-d-glucan in both waste management and power generation industries. Four power plants were studied; one was a dedicated wood pellet boiler, while the other three co-fired a number of different biomass types with coal. A wood pellet manufacturer was also included in the study. Large variations in exposure were observed both between and within worker tasks. The highest average exposures to inhalable dust, (1→3)-β-d-glucan, and endotoxin occurred during wood pellet production (9.6 mg/m^3^ inhalable dust, 12.07 µg/m^3^ glucan and 200 EU/m^3^), but exposures of up to 2104 EU/m^3^ and 290.9 µg/m^3^ glucan were seen in the power plants. Average levels of endotoxin and glucan were lower in the co-firing plants than in the dedicated biomass plant (26.1 EU/m^3^ *vs.* 32 EU/m^3^ and 2.1 µg/m^3^ *vs.* 8.4 µg/m^3^ glucan) but inhalable dust levels were higher (1.3 mg/m^3^ *vs.* 0.48 mg/m^3^).

Madsen *et al.* [99] reported a significant inflammatory response among mice exposed to airborne dust collected from either a combined straw-feeding/boiler room (termed the “boiler room”) or a combined straw-receiving/storage hall (termed the “straw storage hall”). Mice were exposed via intratracheal instillation to either a single dose of dust (18 or 54 µg) from either the boiler room or straw storage hall, or four doses (each 54 µg) on consecutive days. The greatest inflammatory responses were observed in the mice exposed to dust from the straw storage hall, including 30 to 60-fold elevations in mRNA expression in lung tissue for interleukin-6 (IL-6), monocyte chemoattractant protein-1 (MCP-1), and macrophage inflammatory protein-2 (MIP-2) compared to controls. Levels of mRNA for these cytokines were increased about 10-fold in mice exposed to dust from the boiler room. The study authors hypothesized that the inflammatory response was linked with microbial components in the dust, which were generally present at higher concentrations in the dust from the straw storage hall than from the boiler room. Importantly, the study reported a lack of significant increases in DNA strand breaks in bronchoalveolar lavage (BAL) samples, and thus no evidence of DNA damage, for either dust type. Madsen *et al.* [99] cautioned that “more data are needed for an understanding of how the data should be interpreted in a comprehensive risk assessment of exposure at biofuel plants.”

Cohn *et al.* [23] characterized PM components from source biomass (straw and wood pellets), including microbial components and mutagenic activity, from area-level particle samples at the same Danish plant. PM generated via agitation from the source material (pre-combustion) was larger in diameter than PM collected within the facility from the straw storage hall and the boiler room, the latter of which likely included post-combustion ash. Particle diameter ranged from 3.5–5 µm for pure straw biomass, 5.0–7.5 µm for wood pellet samples, and 0.77–0.97 µm for samples from the area of the biomass facility. A large portion of the biomass facility PM was of respirable size, but less so for the raw samples (30%–58% *vs.* 98%). A number of different factors were identified as potential contributors to this difference, including the greater distance from source to measurement in the biomass plant allowing the larger particles to sediment and the presence of combustion PM from biomass and vehicle emissions. PM generated from the raw biomass differed from facility biomass in terms of composition, reactivity (generation of reactive oxygen species), and mutagenicity (Salmonella mutagenicity assay). Specifically, biomass facility PM samples were higher in metal content, polyaromatic hydrocarbons, reactivity potential, thermophilic bacteria (actinomycetes), fungi (*A. fumigatus*) and mutagenic activity, as compared to source-specific biomass generated PM. However, facility samples were collected using a high throughput area sampler potentially contaminated with additional exposure sources (e.g., diesel fumes or other vehicular emissions, welding fumes, *etc.*). As reported by Madsen [20], facility area samples related to straw handling or storage did have high levels of fungal spores and endotoxin, again raising concerns that high pre-combustion exposure may put workers at risk for irritation or inflammatory responses [100,101].

A recent published report from Denmark [102] investigated bioaerosol exposure levels in relation to respiratory symptom and asthma prevalence at a straw and wood chip-fueled power plant. The worker population was compared to a similar occupational group at a more conventional fuel facility. No increased prevalence of pneumonitis symptoms was observed among the biomass facility workers; however, higher asthma symptoms were reported among non-smokers exposed to straw (OR 7.6, 95% C.I. 1.4–12.8) and to lesser extent wood chips. A logistical data analysis reported increased asthma symptoms and work-related respiratory symptoms related to increased endotoxin exposure (OR 1.5, 95% C.I. 1.1–44.4). No statistical associations with endotoxin exposure were observed for rhinitis, conjunctivitis, current asthma, coughing, flu-like symptoms, or diarrhea. Similar associations appeared to be related to fungal exposure. No associations were found between lung function indices and bioaerosol exposure indicators. Albeit a cross-sectional study which lacks the ability to demonstrate causality, this first study adds to the knowledge of exposure methodology, measured levels of bioaerosols, and respiratory symptoms in an industry-specific cohort.

#### 4.1.2. Wood Dust

Wood dust has been recognized as an irritant, sensitizer, respiratory toxicant, and, for a limited number of species, a potential carcinogen [103,104]. The UK Government’s Health and Safety Executive (UK HSE) has also issued guidance on the health risks associated with particular species, as shown in Table 7 [105]; this information was targeted primarily at the wood-working industry and so contains a large number of “unusual” woods, but some species also have relevance in the biomass power generation industry. Mandatory or recommended OELs have been established in a number of regions, including Europe, Canada, and the United States, based on either total inhalable or respirable wood dust, with some authorities specifying lower limits for some wood groups, based on their carcinogenic or allergenic potential, as shown in Table 8 [106,107]. Although the current European Union OEL for hardwood dust is 5 mg/m^3^ of inhalable dust, the EU’s Scientific Committee for OELs has reported that exposure to wood dust at levels of 0.5 mg/m^3^ can induce measurable health effects in the human respiratory system [108]. In a small Swedish study, worker exposure to wood dust ranged from 0.16 to 19 mg/m^3^ (total dust) in a wood pelleting facility, with levels varying across the processing facility [26]. Many of the levels observed were higher than the concurrent Swedish OEL and some studies in other Swedish woodworking industries [103,109]. In a more detailed follow up study by Hagstrom *et al.* [5,24], 35% of inhalable dust samples were above the Swedish OEL. Additionally, larger variation existed between shifts *versus* between workers, indicating that day-to-day temporal variation was higher than inter- individual worker variability.

**Table 7:** Reported health effects associated with wood species (adapted from [105]). Species in bold are known to be in current use as biomass fuels.

Wood Name	Classification	Reported Health Effects
Abura/bahia	Hardwood	vomiting
Afrormosia	Hardwood	skin irritation, splinters go septic, nervous system effects
Afzelia/doussie	Hardwood	dermatitis, sneezing
Agba/tola	Hardwood	skin irritation
**Alder**	Hardwood	dermatitis, rhinitis, bronchial effects
Andiroba/crabwood	Hardwood	sneezing, eye irritation
**Ash**	Hardwood	decrease in lung function
Avodire	Hardwood	dermatitis, nose bleeds
Ayan/movingui	Hardwood	dermatitis
Basralocus/angelique	Hardwood	general unspecific effects
**Beech**	Hardwood	dermatitis, decrease in lung function, eye irritation (possibly from bark lichens)
**Birch**	Hardwood	dermatitis on sawing lumber
Bubinga	Hardwood	dermatitis, skin lesions possible
Cedar of Lebanon	Softwood	respiratory disorders, rhinitis
Cedar (Cent/S American)	Hardwood	allergic contact dermatitis
Cedar (Western Red)	Softwood	asthma, rhinitis, dermatitis, mucous membrane irritation, central nervous system effects
Chestnut (sweet)	Hardwood	dermatitis (possibly from bark lichens)
**Douglas fir**	Softwood	dermatitis, splinters go septic, rhinitis, bronchial effects
Ebony	Hardwood	mucous membrane irritation, dermatitis, possibly a skin sensitizer
Freijo/cordia	Hardwood	possibly a skin sensitizer
Gaboon/okoume	Hardwood	asthma, cough, eye irritation, dermal effects (hands, eyelids)
Gedu nohor/edinam	Hardwood	dermatitis (rare)
Greenheart	Hardwood	splinters go septic, cardiac and intestinal disorders, severe throat irritation
Guarea	Hardwood	skin and mucous membrane irritation
**Gum (southern blue)**	Hardwood	dermatitis
Hemlock (western)	Softwood	bronchial effects, rhinitis
Idigbo	Hardwood	possible irritant
Iroko	Hardwood	asthma, dermatitis, nettle rash
Larch	Softwood	nettle rash, dermatitis (possibly from bark lichens)
Limba	Hardwood	splinters go septic, nettle rash, nose and gum bleeding, decrease in lung function
Mahogany	Hardwood	dermatitis, respiratory disorders, mucous membrane irritation
Makore	Softwood	dermatitis, mucous membrane and respiratory tract irritation, central nervous system and blood effects
Mansonia	Hardwood	splinters go septic, skin sensitization, irritation, respiratory disorders, nose bleeds, headache, cardiac disorders
Maple	Hardwood	decrease in lung function
Meranti/lauan (various)	Softwood	skin irritation
**Oak (various)**	Hardwood	asthma, sneezing, eye irritation
Obeche	Softwood	skin and respiratory tract irritation, nettle rash, dermatitis (handling articles), feverish, sneezing, wheezing
Opepe	Hardwood	dermatitis, mucous membrane irritation, central nervous system effects (e.g., giddiness, visual effects), nose bleeds and blood spitting
Padauk	Hardwood	species-dependent: itching, eye irritation, vomiting, swelling (e.g., eyelids)
Peroba	Hardwood	skin and mucous membrane irritation, systemic effects (e.g., headache, nausea, stomach cramp, weakness), blisters
**Pine (many species)**	Softwood	skin irritation (may cause photosensitization) decrease in lung function
**Poplar**	Hardwood	sneezing, eye irritation, may cause blisters
Ramin	Hardwood	dermatitis (possibly from bark)
Rosewood (many species)	Hardwood	dermatitis, respiratory disorders. Effects may arise from handling wood
Sapele	Hardwood	skin irritation
**Spruce (several species)**	Softwood	respiratory disorders, possible photosensitization
Teak	Hardwood	dermatitis (potent, even after seasoning), nettle rash, respiratory disorders
Utile	Hardwood	skin irritation
Walnut (not African)	Hardwood	sneezing, rhinitis, dermatitis from nut shells and roots
Wenge	Hardwood	splinters go septic, dermatitis, central nervous system effects (e.g., giddiness, drowsiness, visual disturbance), abdominal cramps
Whitewood (American)	Hardwood	dermatitis

The link between wood dust exposure and nasal cancer has been explored in a number of studies, led by Macbeth [110] and Acheson *et al.* [111], with a number of studies in the 1980s and 1990s providing evidence of a relationship between wood dust and sinonasal adenocarcinoma (e.g., [112,113,114,115,116,117]). In 1995, IARC issued guidance that “there is sufficient evidence in humans for the carcinogenicity of wood dust”, with a clear association between adenocarcinoma of the nasal cavities and paranasal sinuses and exposure to hardwood dust [104]. A link with softwood dust is less clear. Identification of specific wood species implicated is problematic, since most of the research has been based on the lumber and furniture making industries, where exposure to a variety of tree species is likely. In Germany, dusts from beech and oak have been classified as carcinogenic since 1985 [118]. Links between wood dust exposure and other cancers are less conclusive, although studies have also indicated higher rates of lung, nasal cavity, nasopharynx, larynx, and prostate cancers with exposure to wood dust, particularly hardwood dusts [117,119,120,121].

**Table 8:** Occupational exposure limits (legal and recommended) for biomass-relevant substances in various countries.

Country/Region	Dust Type	Limits mg/m^3^	Additional comments	Health Endpoint/Comments
		Short-term (15 min)	Long-term (8 h. Time Weighted Average)	
*Wood dusts*				
US (OSHA)	Particulate not otherwise regulated (includes wood dust)—inhalable—respirable		15 5	Throat, skin, eye irritation, upper respiratory problems
US (NIOSH recommended)	Wood dust		1	Pulmonary Function, Carcinogen
European Union (applies to all member countries)	Hardwood (inhalable fraction)		5	Carcinogenic, sensitizer
UK	Softwood (inhalable fraction)		5	Sensitizer
Australia	Hardwood		1	
Australia	Softwood		5	
Ontario, Canada	Certain hardwoods such as beech and oak		1	
Ontario, Canada	Softwood	10	5	
Sweden	Inhalable non-impregnated wood dust		2	Carcinogen
Sweden	Impregnated wood		0.05	Applies if levels of impregnating substances (with their own OELs) are unknown
Australia	Softwood	10	5	Sensitizer
Australia	Certain hardwoods such as beech and oak		1	Sensitizer
Germany	Respirable wood dust		2	Selected species identified as carcinogenic and/or sensitizing
Russia	Wood dust		6	Maximum allowable concentration, sensitizer, fibrogenic action
US (OSHA/California)	Wood dust, all soft and hard woods except Western red cedar	10	5	
US (OSHA/California)	Wood dust, Western red cedar		2.5	
*Other biomass dusts*				
US (OSHA)	Grain dust (oat, wheat, barley)		10	
UK	Grain dust (inhalable fraction)		10	Sensitizer
*Trace metals in biomass ash*				
UK	Cadmium and Cadmium compounds (as Cd)		0.025	Carcinogenic (selected compounds)
UK	Cobalt and Cobalt compounds (as Co)		0.1	Carcinogenic (selected compounds), sensitizer
UK	Manganese and inorganic manganese compounds (as Mn)		0.5	
US (OSHA)	Cadmium dust	0.5	0.2	
US (OSHA)	Cobalt metal, dust, and fume (as Co)		0.1	
US (OSHA/California)	Cadmium		0.005	
US (California)	Manganese and compounds, as Mn		0.2	
US (OSHA/California)	Cobalt metal, dust, and fume (as Co)		0.02	

The association between exposure to wood dust and asthma symptoms was reported in the 1940s [122] and has repeatedly been identified since (e.g., [123,124,125,126]). A meta-analysis of the data by Pérez-Rios *et al.* [127] suggested that exposure to wood dust could increase the risk of work-related asthma by 50%. In a number of these studies, sensitization to specific wood species has been identified. In the De Zotti and Gubian  study [123], bronchial provocation tests identified obeche, chestnut, acacia, and iroko woods as being likely to cause asthma symptoms in four cases, while oak, beech, and pine woods triggered rhinitis in three cases. Positive responses to respiratory provocation and skin challenge tests and specific IgE antibodies to ash wood were found by Fernández-Rivas [124] in the case of a furniture factory worker suffering rhinitis and asthma symptoms. In the UK, physicians report cases of occupational asthma to the SWORD database [128], with estimates given for the number of diagnoses linked to different potential causative agents. Between 1998 and 2012, wood dust was ranked as the third most common causative agent in terms of the average number of cases of occupational asthma linked to exposure reported each year (15 cases), behind isocyanates (49 cases) and flour (29 cases). Note that incidence rates are not reported for individual causative agents. As these data rely on both a positive diagnosis of occupational asthma and identification of the causative agent by a respiratory specialist, these figures may be underestimated.

#### 4.1.3. Volatile Organic Compounds (VOCs)

Monoterpenes, such as α-pinene, β-pinene, and Δ^3^-carene, are derived from biomass and may be of concern due to their ability to irritate eyes, skin, and mucous membranes [26,109]. The previously mentioned small study among Swedish wood pellet workers (5) reported personal exposures to monoterpenes in the range of 0.64 to 28 mg/m^3^, below the Swedish occupational exposure limit of 150 mg/m^3^ (total or individual monoterpene, 8-h TWA. However, intercompany variation appeared to be substantial, potentially due to variable moisture content or wood type. The correlation between wood dust and monoterpenes was moderate (0.44). A related concern has been raised regarding oxidization of monoterpenes to form both particle- and gas-phase reaction products that may induce respiratory effects [129].

In the Swedish wood pellet production study [5] measurements also included resin acids, α-pinene, and total VOCs. Resin acids ranged from <0.33–10 µg/m3 and α-pinene from <0.23–25 mg/m3 (β-pinene and Δ3-carene were below detection limits for the majority of samples). Workers were exposed to multiple resin acids; although no OELs exist for these compounds, exposure in other industries to colophony (a resin-containing compound also known as rosin, and used in soldering flux, adhesives, and polishes among other products) has been associated with occupational asthma and contact dermatitis [130]. The correlation between total dust and resin acids was moderate. Monoterpene levels varied by location and relative age of the raw material, with newer raw material associated with higher measurements. The VOC analysis identified a range of compounds including terpenes, C6-C11 aldehydes (e.g., hexanal, heptanal and nonanal), and other hydrocarbons (e.g., ethylacetate, propionic acid, 1-pentanol, and 2-butanone), all of which have been identified as irritant chemicals [131].

Svedberg et al. [28] investigated levels of a number of organic gases during storage at three pellet production plants, both in warehouses containing pellets and in domestic storage rooms. The principle organic compounds identified at two of the warehouses were aldehydes (50%–60% w/w), acetone (30%–40% w/w), and methanol (10% w/w) (the third warehouse had an ambient temperature of −10 °C and levels were below detection limits). In one warehouse, a peak aldehyde reading of 457 mg/m3 was recorded at the surface of the pellet pile, with hexanal (70%–80%wt) and pentanal (10%–15%wt) predominating. Auto-oxidation of fatty acids in the wood was proposed as the mechanism of formation of these compounds, the rate of which increases with temperature. Hexanal and carbon monoxide were also present in the emissions from pine lumber drying at these plants. Human exposure studies indicate that hexanal concentrations of 10 ppm are sufficient to invoke symptoms of mild irritation [132].

#### 4.1.4. Carbon Monoxide (CO)

Carbon monoxide exposure, such as that which could occur in confined or enclosed spaces where wood pellets are stored or transported, has resulted in accidents and fatalities [28,133]. In the Hagstorm *et al.* study [5], all CO levels remained below 1.6 mg/m^3^. In an earlier Swedish report, air sampling in one warehouse containing freshly produced wood pellets showed CO levels of 54 mg/m^3^ at the ceiling [28]. In 2012, Gauthier *et al.* reviewed the deaths of two people, one in Germany in 2010 and the other in Switzerland in 2011, both of which were linked to CO exposure in storage rooms of multi-household wood pellet heating systems [30]. These systems consist of an airtight storage room (filled pneumatically from the outside) which feeds a boiler supplying hot water to the surrounding houses. In normal operation, the storage room is not entered—both casualties were investigating faults in the pellet handling system at the time. In the Swiss case, CO measurements of 7500 ppm were recorded several days after the event, with 2 h of ventilation only reducing this to 2000 ppm (note the system guidelines recommended 15 min of ventilation prior to accessing the storage area). Subsequent experiments confirmed that the CO was likely generated from the wood pellets rather than a fault in the combustion system, and that the area was also likely to be oxygen deficient. A third fatality was reported in Ireland after a householder entered his 7-ton capacity pellet storage room [134]. Two deaths in different wood pellet silos have been reported in Finland [30]. Ship holds appear to be particularly susceptible to the buildup of lethal levels of CO, coupled with oxygen depletion, with six deaths during wood pellet transport and at least three during transport of other woody materials reported since 2002 [27,28,29,30,135]. This is most likely due to the gradual decomposition of biomass and release of CO and CO_2_ [136]. High oxygen and temperature can accelerate this process [27].

Emissions from off-gassing have also been recorded during storage of non-pelletized wood material. He et al. [137] stored logging residues in sealed containers at 15 °C and 35 °C. At 35 °C, after 10 days, oxygen levels in the headspace of the containers had decreased to near-zero, while CO2 was present at 13.8%, CO at 0.16%, and CH4 at 0.15%. Also detected in the headspace were numerous volatile organic compounds (total concentration 85 ppm), including alcohols, aldehydes, acids, acetone, benzene, ethers, esters, and terpenes. High product turnover, good ventilation and high oxygen levels may be expected to decrease the likelihood of off gassing [27]; however, at biomass-fueled generation facilities a number of different storage systems are in use, and so the effectiveness of these cannot be guaranteed. Biomass may also be stored for significant periods of time, e.g., during an unplanned shutdown, which increases the risk of off-gas accumulation.

### 4.2. Combustion-Associated Risks

Occupational studies focusing on the potential health risks posed by exposures to biomass combustion products at large scale biomass power plants are lacking, with minimal data on potential SSHs and their exposure levels relative to facility, worker tasks, working environment, and biofuel stock. Because there remains uncertainty regarding the specific components, concentrations, and related nature of the health risks posed by specific biomass combustion products from modern power plants, this section must rely on identifying the potential for adverse OH&S effects based on data from other biomass exposures scenarios, including poorly- or uncontrolled biomass combustion such as wildfires. However, given the greater degree of control over both combustion quality and specific pollutants as well as the high level of dispersion from the stacks of utility scale power plants, data from these less-controlled sources should be considered worst-case. Although occupational exposure of power plant workers to combustion gases is expected to be low during normal operation, self-heating and spontaneous combustion of stored biomass (due to biological and chemical oxidation reactions) is a recognized issue in many industries, including the power sector [9]. Under these situations, there is a risk of worker exposure to products from incomplete biomass combustion and smoldering. However, the frequency of such exposures is expected to be low and the size of the affected population limited.

#### 4.2.1. Health Effect Studies of Relevance and Uncertainties in the Available Studies

A wide range of literature exists on exposure to smoke from residential wood burning, prescribed burning, and wildfires, as well as resultant health effects (see reviews by e.g., [6,138,139]). Therefore, this section relies on data from these alternate combustion technologies (e.g., small domestic woodburning appliances such as woodstoves, wood log boilers, and fireplaces, and also forest and brush fires) to explore the potential health risks posed by the occupational exposure to biomass combustion products within commercial biomass power plants.

It is important to emphasize the large uncertainties associated with the consideration of health effects data from these studies. Major factors leading to differences in occupational exposures at power plants *versus* uncontrolled ambient exposures include variability in the composition and physicochemical properties of the combustion gases from biomass, and thus the potential toxicity of the mixture, based on biomass fuel type and properties, boiler type, and combustion conditions. In particular, the completeness of the combustion process is a key determinant of the levels and composition of biomass emissions [140]. In general, concentrations of CO, VOCs such as acrolein, formaldehyde, and benzene, gaseous and particulate PAHs, and other organic species are enriched in emissions from incomplete biomass combustion [141]. With incomplete combustion, particle emissions are dominated by condensable organic particles, soot, and char [33]. In contrast, large-scale boilers, representative of a modern biomass-fueled power plants, generally operate under more controlled and stable combustion conditions that favor quasi-complete combustion [33,138]. Under such optimal combustion conditions, organic carbon content of particle emissions can be negligible, and inorganic ash can dominate particle emissions [33,140]. Combustion conditions are more variable in domestic wood-burning appliances such as woodstoves and fireplaces, typically yielding emissions rich in both soot and organic carbon particles and containing lesser amounts of inorganic ash [140]. Incomplete combustion, and thus emissions dominated by organic carbon particles and hydrocarbons, appear more prevalent for prescribed burning and wildfire events where low temperatures and smoldering conditions prevail [140,142].

Another source of information on the impact of biomass combustion on health is research on household biomass combustion for heating and cooking purposes in developing countries. These studies illustrate the significant public health burden of indoor biomass combustion in these populations, including an estimated 1 to 2 million premature deaths per year due to chronic obstructive pulmonary disease (COPD), acute and chronic respiratory disease, tuberculosis, and lung cancer [6]. Given that unvented stoves continue to have widespread usage in developing countries, often discharging emissions directly into the living space, such exposures and related health risks for these biomass combustion scenarios are unlikely to reflect occupational exposure at electricity-generating biofuel power plants. Therefore, particularly large uncertainties exist regarding the relevance of health effects findings from studies either based on wildfires or prescribed agricultural or wild land burning or from studies of the effects of indoor household combustion relative to biomass combustion sources favoring more efficient and complete combustion, such as modern biofuel plants. Nonetheless, despite these uncertainties, findings from such epidemiologic studies do have the statistical power to detect possible biomass combustion product-related health outcomes.

Finally, given the scarcity of relevant studies, both on health effects and exposure data specific to occupational environments at biofuel plants, it is not feasible to quantify biomass-specific risks posed to workers. Instead, assessments of potential adverse occupational effects are currently limited to qualitative extrapolation of findings from controlled exposure studies and epidemiologic studies in populations exposed to uncontrolled biomass smoke. Furthermore, while experimental animal data exist describing the toxicity of various types of biomass combustion products (as reviewed by [6,139]), until better sector-specific occupational exposure characterization becomes available for biofuels of concern, these data are of limited quantitative value.

#### 4.2.2. Studies of Occupational Exposures and Potential Health Risks at a Large-Scale Danish Biofuel Plant

To date, only the research group based at the Danish National Research Centre for the Working Environment has reported on potential occupational exposures and related toxicities at a large-scale biofuel plant, specifically a straw-fueled 8.3 MW electricity-generating facility in Zealand, Denmark [23,25]. Studies have addressed pre-combustion emissions such as organic dust and bioaerosols (e.g., [20,25,95]), with more recent limited data related to combustion-related PM [23].

As discussed earlier, Cohn *et al.* [23] investigated the mutagenicity and generation of highly reactive oxygen species (hROS) of respirable PM samples collected from the boiler room of a Danish biofuel facility (as well as PM samples reflecting pre-combustion materials from the straw storage hall at the same facility, as well as test samples of biomass-derived PM obtained by placing straw (*n* = 9) and wood pellets (*n* = 1) in a rotating drum). Using a *Salmonella* mutagenicity assay, they reported evidence of mutagenicity for the majority of the PM samples collected from the boiler room. In addition, they observed higher hROS generation in a cell-free chemical assay for the boiler room PM samples than for the biomass stock-derived PM samples. As discussed by the study authors, these findings suggest greater biological activity of biomass-combustion PM *versus* biomass-derived PM (e.g., pre-combustion biomass fuel), although they note that boiler room PM likely consists of a complex combustion mixture of both biomass and vehicular emissions (e.g., trucks and diesel-powered forklifts), thereby limiting the biomass-specific apportionment of both exposure and risk. Overall, these findings provide limited evidence of the potential toxicity of biomass combustion PM from a modern biofuel facility, primarily using straw-based stock fuel. Additionally, the limited number of sites sampled indicates the potential difficulty in apportioning the portion of combustion PM due to biomass stock or other facility sources.

#### 4.2.3. Controlled Human Exposure Studies of Small-Scale Biomass Combustion

Controlled exposure studies, or chamber studies, are considered to provide some of the more useful data for assessing the potential health risks of inhaled pollutants due to the use of human subjects, well-defined exposure concentrations and durations, and precise measures of biological responses [143]. As summarized in Table 9, a number of controlled exposure studies have utilized biomass smoke generated from domestic wood burning appliances, including woodstoves and wood pellet boiler systems. These small combustion appliances are less efficient and more poorly controlled than large boilers in biomass fueled power plants, thereby contributing to differences in emissions (e.g., higher organic carbon and soot content) relative to biofuel plants, as discussed previously. As a result, these results are only briefly discussed and reference is made to the source material cited for further details of the testing undertaken.

Despite the use of highly elevated exposure levels of biomass smoke, as reflected by PM_2.5_ concentrations in the range of 150 to >600 µg/m^3^, these studies have generally reported evidence of fairly mild and readily reversible biological responses (Table 9). Observed effects include relatively small increases in some biomarkers of lung and systemic inflammation, airway oxidative response, blood coagulation response, or lipid peroxidation, including changes in several biological markers achieving statistical significance. As shown in Table 9, many of these studies reported inconsistent findings for some types of biological responses, or a greater number of negative findings (*i.e.*, no changes compared to control) than statistically significant positive findings.

Human controlled exposure studies of healthy adult volunteers thus provide some evidence of statistically significant, but generally mild, biological responses to elevated smoke exposures from uncontrolled biomass combustion, in particular lung and systemic inflammation and an airway oxidative response. While the physiological significance of some of the observed responses is ambiguous, they provide evidence of potential respiratory and cardiovascular health risks from elevated exposures to biomass smoke. However, it is again important to emphasize the uncertainties regarding the relevance of these findings to the biomass combustion gas at modern biofuel plants.

#### 4.2.4. Epidemiologic Investigations of Uncontrolled Ambient Biomass Smoke

Epidemiologic studies of populations affected by biomass smoke are more numerous than human controlled exposure studies. None are currently available for workers or communities impacted by biomass combustion emissions from a modern biofuel plant, and findings from the available studies of wildfires and prescribed burning are of uncertain relevance to occupationally exposed workers at modern biofuel plants due to potential differences in combustion conditions and the properties of combustion emissions (as discussed earlier). In addition, it is important to note that epidemiologic studies have a variety of other general limitations and uncertainties that contribute to the difficulty in making causal conclusions based on this type of health effects evidence only, including model selection and specification, treatment of co-pollutants, control of potential confounders (e.g., smoking, seasonal effects), and exposure misclassification. A particular advantage of epidemiological studies compared to human controlled exposure studies, however, involves their frequent study of large populations and thus increased statistical power to detect rare health outcomes.

Overall, epidemiological findings regarding smoke from uncontrolled biomass combustion are mixed. Table 10 summarizes the epidemiologic literature related to short-term studies of exposures to biomass smoke in areas impacted by large-scale biomass combustion events. These studies show a range of outcomes, from increased emergency department visits to mortality. There is now a consistent body of epidemiologic evidence linking elevated short-term exposure to biomass smoke with increased risk of a variety of respiratory-related health impacts. Despite a growing number of studies, there is little epidemiologic evidence linking biomass smoke exposure to either cardiovascular-related health outcomes or mortality. In addition, the epidemiologic evidence linking biomass smoke exposure and cardiovascular health outcomes is significantly weaker than that linking urban PM_2.5_ with cardiovascular morbidity and mortality [144].

#### 4.2.5. Regulatory Consideration of Biomass Combustion Emissions and Cancer Risk

IARC [145] has classified indoor emissions from household biomass combustion (primarily wood) as “probably carcinogenic to humans” (Group 2A). In its report, IARC cited limited evidence in humans and experimental animals for the carcinogenicity of household biomass combustion emissions, but sufficient experimental evidence in animals for the carcinogenicity of wood smoke extracts. Indeed, the mutagenic potential of biomass smoke PM extracts is well documented in both bacterial systems and human and animal cell lines [6,140,146]. Study findings suggest relationships between mutagenic activity and a number of factors, including combustion combustions, type of wood-burning device, fuel type and origin, and PAH content [6,140,147]. In particular, Klippel and Nussbaumer [147] reported greater evidence of chromosome aberrations in a micronucleus test of a Chinese hamster lung fibroblast cell line for particles generated during incomplete combustion conditions than for more complete combustion conditions, where the number of chromosome defects was below the limit of detection. Mixed evidence of the carcinogenicity of wood smoke is available from laboratory animal studies [6].

Epidemiologic studies investigating the health impacts of long-term exposures to biomass combustion emissions in developed countries are limited, and thus large uncertainty exists regarding cancer risk posed by combustion product mixtures relevant to modern biofuel plants. Some studies have reported significant associations between ambient fine PM (PM_2.5_) and increased cancer risk, in particular lung cancer (e.g., [148]). However, biomass combustion emissions are generally a relatively minor contributor to ambient PM_2.5_ in the urban locations included in these studies compared to other PM_2.5_ sources such as traffic emissions and coal-fired power plant emissions, and these findings are thus of uncertain relevance to specific PM types such as wood smoke PM.

#### 4.2.6. Conclusions Regarding the Evidence for Biomass Combustion Product Health Risks at Large-Scale Modern Biofuel Facilities

Although the specific magnitude of any potential human health risk is a function of a variety of factors, multiple lines of evidence suggest that short-term exposure to elevated levels of biomass combustion products could increase the risk of respiratory-related health impacts. There is large uncertainty associated with potential long term effects. However, the probability of such exposure among workers at biofuel plants is expected to be low). Furthermore, there are significant compositional differences between emissions from modern biofuel plants and the biomass combustion sources that have been the focus of the bulk of the health effects research (*i.e.*, woodstoves, fireplaces, forest and brush fires). Combustion of biomass produces a complex mixture, and there is significant toxicological information on many of the individual constituents, including criteria pollutants, several different classes of VOCs (e.g., acrolein, aldehydes) and some associated persistent semi-volatile compounds (e.g., PAHs, dioxins/furans). However, it must be emphasized that in the absence of reliable monitoring and exposure estimates, it is uncertain if these constituents would be present at levels within biomass plants that could cause health effects in workers.

There is even more uncertainty associated with cardiovascular health impacts and mortality. As mentioned previously, numerous epidemiological studies have reported associations between cardiovascular morbidity and mortality and ambient PM_2.5_. Moreover, there is accumulating evidence on the role played by specific PM components in adverse health effects, with some indication that carbonaceous PM (*i.e.*, elemental and organic carbon) may play a larger role than other constituents (e.g., [149,150]), and others suggesting the importance of certain trace metals and other components [151,152]. However, despite some similarities in composition compared with other types of combustion emissions, PM from biomass combustion can have very different composition and physicochemical properties, and thus potentially differing toxicity [6,140,153,154]. Some studies (e.g., [147,155,156]) suggest that biomass combustion PM, and in particular that associated with quasi-complete combustion in well-operated boilers, is of lesser toxicity than other types of combustion emissions. In contrast, other studies (e.g., [142,157]) reported findings indicating that biomass combustion products, in particular those from forest fires and prescribed fires, are of similar—if not greater—toxicity than other types of combustion emissions.

Another area of uncertainty relates to discrepancies between environmental and occupational epidemiology studies of PM exposure. In contrast to the number of epidemiologic studies that have reported statistically significant associations between ambient PM_2.5_ and increased risk of mortality in the general population, many large occupational epidemiologic studies (e.g., [158,159,160]) have failed to observe increased mortality risk among worker populations with highly elevated PM exposures, including workers in the carbon black industry who are routinely exposed to combustion emissions.

### 4.3. Post-Combustion Risks

Studies of health effects in workers exposed to biomass ash in power generation facilities are limited. From studies in workers that handle coal ash, however, it is known that key hazards for conventional ash exposure relate to the potential inhalation of PM and trace inorganic compounds (e.g., arsenic, chromium, cadmium) [161]. Also of potential concern are free respirable quartz [162,163] and radiological exposures [163]. It should be noted, however, that even if these properties of coal ash pose a potential concern, evidence from epidemiological, animal, and *in vitro* studies, albeit limited, supports the conclusion that coal ash exposure is not associated with silicosis [162,164]. Concerns related to potential exposures to organic compounds (e.g., dioxin, PAHs) in coal ash have also been raised, but these levels have been repeatedly shown to be close to detection limits [165,166] Potential routes of exposure to biomass-derived ash are expected to mimic those of coal ash, with inhalation of PM and associated compounds being the primary concern. Additional exposure could occur via dermal contact or ingestion if hygiene measures are inadequate to prevent contamination of welfare areas.

#### 4.3.1. Ash and Inorganic Compounds

To understand potential health risks from exposure to biomass ash it may be informative to compare the ash generated by coal and biomass combustion. Ash from solid fuel combustion consists of a mixture of the inorganic components of the fuel and unburnt carbon. It may also contain materials added to assist in the combustion process, such as the bed material (typically sand) used in fluidized bed combustion, or materials to control pollutant emissions (e.g., limestone for acid gas control). At the industrial scale, the amount of unburnt carbon in the ash is minimized, so the major component of the ash is the mineral matter contained in the fuel. While both biomass and coal can vary considerably, most biomass is lower in ash than most coal. The mineral composition of the ash also varies significantly by fuel source and combustion process [167]. Van Loo and Koppejan [9] reported that ash levels varied from about 0.5 wt% to 12% (on a dry basis), with hardwood, straw, and wood contaminated with inorganic impurities on the higher end of the range. Nonetheless, despite the lower ash content of these fuels, ash is a major contributor to overall dust loads at biomass power generation facilities and thus can constitute risk related to PM inhalation. In addition, although the total ash content of biomass is usually less than coal, the water-soluble fraction (including compounds of alkali and alkali earth metals) can be higher [9]. Chemical analyses of different fly ash size fractions at biomass-fired plants have shown that these alkali metal compounds form fine particles in the flue gas [168]. The health implications of this are not well studied, but by implication, the levels of these alkali metals in the fuel could affect the emission rate of respirable ash particles.

Under Europe’s Registration, Evaluation and Authorization and Restriction of Chemicals (REACH) regulations, biomass ash has been registered as a UVCB substance (unknown or variable composition, complex reaction product or biological origin), with identified components including oxides of calcium, sodium, potassium, silicon, iron, manganese, magnesium, aluminum, phosphorous, titanium and sulfur [69]. The associated chemical safety assessment concluded that biomass ash did not require hazardous classification under REACH. A significant amount of work has been undertaken to characterize “clean” biomass ash, primarily from wood-fired boilers in Scandinavia, where the use of such ash as a forestry fertilizer is permitted (see for example [170]). Various studies and databases have complied data on the macro and trace element composition of biomass ash. Data from a number of sources are presented in Table 11 and are compared to these same trace metals measured in coal ash and soil. Someshwar [171] compiled information on the trace metal content of wood ash collected from a variety of sources (26 ash samples in all). Most of the ashes were from pulp mill bark boilers, although ash samples from other types of large capacity wood boilers are represented. In addition to the Someshwar analysis, the International Energy Agency has collected information on the trace metal content of biomass ash produced from different processes and fuels. The database currently includes 560 ash samples from biomass burning facilities with capacities from 400 kWth to 6.3 MWth in six different countries, although many of these samples were only analyzed for a limited number of elements. In Europe, data on wood ash composition have been collated by the Swedish University of Agricultural Sciences [170] and The Energy Centre of the Netherlands [10], although much of the data are shared between these databases. In general, the data in Table 11 reflect biomass boilers of different types, sizes and fuels; this is reflected in the wide range of values for most of the trace elements. It is unclear how representative some of these data are for ash from large-scale commercial boilers, although much of the data in the SLU and ECN databases were obtained from dedicated biomass generation plants. For metals in particular, there is a relationship between levels in the fuel and levels in the ash. However, most large boilers have multiple ash streams (e.g., bottom and fly ash), and, as is the case with coal, some metals preferentially condense into certain ash products, resulting in different concentrations in each stream [172]. This is evident in the data from the ECN database, where many of the elements are enriched in the fly ash compared to the bottom ash.

The metal content of ash derived from various fuel sources differs. In general, ash from the burning of straw, cereal, and grasses is lower in metals compared to ash from woody material and bark [9]. As shown in Table 11, average trace metal concentrations in ash from the burning of waste wood (regularly used as a fuel for electricity generation in Europe) are considerably higher than levels in clean wood ash. Compared to coal ash, clean wood ash generally contains lower levels of arsenic, chromium, and nickel. Biomass ash, however, does appear to be enriched in manganese, cobalt, cadmium, and zinc compared to coal ash and soil. However, even when summed they constitute less than 2% of the total ash composition, and so, provided occupational exposure limits for general dust are complied with, exposure to these metals is unlikely to reach the limit values for individual metals.

One experimental study (reported in [173]) involved exposure of rats via inhalation to fine (*i.e.*, equivalent to emitted) fly ash from coal, biomass, and coal/biomass co-firing. No significant impact on lung inflammation was seen with the biomass-derived fly ash compared to titanium oxide control, while coal and coal/biomass ash elicited significant effects, e.g., increases in IL-8 and PMNs. The magnitude of these effects was lower than the effects of carbon black, the positive control. The authors theorized that this may be a result of the higher percentage of soluble salts in the biomass ash; while all the ash samples were of a similar size (mean mass aerodynamic diameter 1.5–3 µm), resulting in similar deposition rates in the lungs, biomass ash could dissolve and be eliminated from the lungs, while the less soluble coal ash remained.

#### 4.3.2. Polycyclic Aromatic hydrocarbons (PAHs)

PAHs form from the incomplete combustion of organic material. Consequently, the combustion of biomass has the potential to generate PAHs, which can adsorb to ash particles and thus become available for oral, dermal, and inhalation exposures; however, bioavailability can be modified by a number of factors [174]. PAH generation is diminished with complete burning (low carbon in ash levels), which would be more characteristic of large commercial biomass boilers used to generate electricity. Interestingly, some of the technologies that reduce NO_x_ emissions in large commercial boilers, such as staged combustion, may also cause PAH formation in ash to increase [175]. Data on the PAH content of biomass ash, including benzo(a)pyrene (often used as a marker for total PAH levels), are limited. The available data show that, in general, the PAHs found in wood ash are two and three-ring compounds, as opposed to the more toxic 4-and 5- ring compounds; naphthalene is the most abundant PAH [171]. Data from some larger scale facilities are presented in Table 12. With the exception of filter fly ash from bark combustion, these PAH levels are within the range of levels found in background urban soils [176]. Like metals, the highest level is associated with the filter fly ash. In conclusion, because high-capacity commercial boilers favor complete combustion conditions, it is unlikely PAHs in ash would be of toxicological concern for utility workers.

#### 4.3.3. Dioxins/Furans

There is substantial information on dioxin levels of biomass ash, but the information mainly comes from small-scale combustion units or uncontrolled burning. Despite the limited information from large-scale facilities, some general principles can be garnered from the available information. Generation of dioxins and furans is favored under conditions where the fuel stock contains higher levels of chlorine. In addition, incomplete combustion is associated with higher levels of dioxins and furans (*i.e.*, higher ash levels are correlated with higher dioxin content). In general, herbaceous materials (straw, cereal) have higher chlorine content compared to wood and bark, and consequently the ash generated is associated with higher levels of dioxin/furans [9]. As with PAHs, while dioxin should be considered as part of a thorough risk evaluation, the concentrations of dioxins and furans in biomass ash (expressed as toxic equivalents of TCDD) are generally within levels found in background soils and below health-screening levels]. Pitman [167] reviewed several available datasets and concluded that the PCDD/F contents of both “domestic” grate ash and “commercial” wood boiler ash are “negligible”. This is especially true of clean wood burned in commercial boilers. However, as reviewed in Someshwar [171], salt-laden wood can generate significantly higher levels, which may be important for fuel harvested from more coastal regions. In addition, the burning of waste wood or residual wood can produce higher levels of PCDD/F in ash than the combustion of clean wood fuel [9] Due to the potential variability in levels of dioxins and furans, biomass combustion facilities would need to undertake ash analysis to understand the potential range of PCDD/F in the ash produced by their boiler/fuel combination to use as the basis of a risk-based assessment.

**Table 9:** Human controlled exposure studies of inhaled woodsmoke biological effects.

Reference	Exposed Population	Combustion Source	Dominating Particle Types	PM_2.5_ Exposure Levels ^1^	Key Statistically Significant Acute Biological Responses ^2^	Key Negative Findings ^2^
[177,178,179,180]	13 healthy adults	Small cast iron wood stove Fuel: Standardized mixture (50/50) of hardwood/softwood (birch/spruce), dried for 1 yr (moisture content 15%–18%) Exposure: 4 h	Organic carbon/soot	240–280 μg/m^3^	↑ Serum amyloid A; ↑ Plasma factor VIII; ↑ Factor VIII/von Willebrand factor ratio; ↑ Urinary excretion of free 8-iso-prostaglandin_2α_; ↑ Malondialdehyde in breath condensate; ↑ Serum Clara cell protein; ↑ FENO_270_ and calculated alveolar NO ↓ PBMC levels of DNA strand breaks; ↑ mRNA levels of hOGG1	“Weak” subjective symptoms; No significant increases in serum C-reactive protein (CRP), fibrinogen, IL-6, or TNF-α levels; No significant changes in RBC, Hb, Hct, leukocytes, or platelets; No significant change FENO_50_ or NO influx; No significant increase in urinary Clara cell protein No significant changes to FPG sites, hOGG1 activity, or PBMC expression of hNUDT1 or HO-1; No significant changes in urinary excretion of 8-oxodG or 8-oxoGua
[181]	10 healthy adults	Electric element in a woodstove Fuel: Red oak wood Exposure: 2 h	Organic carbon/soot	485 ± 84 μg/m^3^	↑ Percentage and absolute numbers of neutrophils in blood, BL, and BAL; ↑ IL-1β in blood; ↑ blood LDH c	No significant changes in symptom prevalence or lung function; No significant changes blood or BAL cytokine concentrations (IL-6, IL-8, TNF-α); No significant changes white blood cell counts, blood coagulation (e.g., von Willebrand’s factor, plasminogen activators) or total proteins and albumin; Minimal changes in cardiac endpoints
[182]	26 healthy adults	Standard woodstove Fuel: Dried pine wood with UV aging woodsmoke Exposure: 3 h	Organic carbon/soot	150–200 μg/m^3^	None	No significant changes in vascular function measured by reactive hyperemia-peripheral arterial tonometry (RH-PAT)
[183]	20 healthy adults	Standard woodstove (operated “optimal conditions”) Fuel: Dried beech Exposure: 3 h	Combination of alkali salts, soot, and organic matter	165–662 μg/m^3^	↑ Self-reported subjective symptoms (significant changes for 5 of 6 indices): “environmental perception” “irritative body perceptions” “psychological/neurological effects” “weak inflammatory” ↑ Self-reported general mucosa irritation	No increase in the index for “lower respiratory effects”
[184]	19 healthy adults	Adjustable wood pellet boiler system (operated under incomplete combustion) Fuel: Moist softwood pellet/sawdust mixture from pine and spruce (18% moisture)	Organic carbon/soot	224 ± 22 μg/m^3^	↑ Glutathione in BAL; ↑ Upper airway symptoms (nose and throat irritation)	No significant changes in lung function (VC, FVC, FEV_1_) or exhaled NO (FE_NO_); No significant changes peripheral blood counts; No significant changes GSH in BW or endobronchial biopsy tissue; No significant changes in lung inflammatory parameters (e.g., MPO, MMP-9), levels of other antioxidants (GSSG, vitamin C, and urate), or enzymes indicative of oxidative stress (HO-1, GST) in BAL, BW, and endobronchial biopsy tissue

**Notes:** (1) Exposures are for whole woodsmoke and thus reflect exposures to not only particulate matter (PM) but also gaseous constituents including NO_x_, CO and a number of gaseous hydrocarbons. The PM concentration is an indicator of the level of exposure; (2) Significant effects reflect significant differences between woodsmoke and clean air exposures; BAL = bronchoalveolar lavage; BL = bronchial lavage; BW = bronchial wash; FENO_50_ = fraction of exhaled NO at a flow rate of 50 mL/s; FENO270 = fraction of exhaled NO at a flow rate of 270 mL/s; FEV_1_ = forced expiratory capacity in one second; FPG = formamidopyrimidine-DNA-glycosylase; FVC = forced vital capacity; GSH = glutathione; GSSG = glutathione disulfide;GST = glutathione transferase; Hb = hemoglobin; Hct = hemocrit; hNUDT1 = nucleoside diphosphate linked moiety X-type motif 1; HO-1 = heme oxygenase 1; hOGG1 = oxoguanine glycosylase 1; IL = interleukin; LDH = lactate dehydrogenase; MMP-9 = matrixmetalloproteinase 9; MPO = myeloperoxidase; NO = nitric oxide; 8-oxoGua = 8-oxo-7,8-dihydro-oxoguanine; 8-oxodG = 8-oxo-7,8-dihydro-2 –deoxyguanosine; PBMC = peripheral blood mononuclear cells; RBC = red blood cells; TNF = tumor necrosis factor; UV = ultraviolet; VC = vital capacity.

**Table 10:** Health outcomes linked with biomass smoke exposure in epidemiologic studies.

Health Outcome	Example Reference(s)
Emergency department (ED) visits for respiratory diseases, including asthma	[185,186,187,188]
Respiratory hospital admissions	[189,190,191,192,193,194]
Respiratory physician outpatient visits	[194,195,196,197]
Respiratory symptoms	[198,199,200,201]
Lung function	[202,203,204,205]
Pulmonary and systemic inflammation	[202,206,207]
Cardiovascular-related health outcomes	Vascular function- 207; ED visits for cardiovascular diseases-208 **^a^**
Mortality	[209,210] **^b^**

**Notes**: **^a^** In general, the epidemiological evidence linking biomass smoke exposure with cardiovascular-related health outcomes is weak and inconsistent, with most pertinent studies failing to observe statistically significant associations [155,188,192,193,194,197,211,212,213,214,215]; **^b^** Most other studies have reported no evidence of an association between biomass smoke and mortality, including [155,193,195,214,216].

**Table 11:** Trace elements measured in biomass ash (number of samples).

	As	Cd	Cr	Pb	Hg	Co	Cu	Mn	Ni	Zn
Median (mg/kg)										
Wood Ash **^a^**	10	3.6	30.8	61.5	0	9	68.2	3485	16.4	329
All Fuels-All Ash fractions **^b^**	9	17	107.5	36	9.5	16	146	14,350	55	1659.5
Wood Chips-All Ash fractions **^b^**	8	19	132	39	10	14.5	180	14,366	55	350
Wood Ash—all boiler types **^c^**	7.98 (558)	8.4 (619)	66.4 (567)	54 (607)	0.11 (549)	10.2 (543)	101 (659)	8200 (551)	33 (563)	1438.5 (656)
Waste Wood-fly ash **^d^**	104	456	404	50,000	<0.5	11	422	na	74	164,000
Coal Ash-Fly Ash **^e^**	71	1.07	133	49	0.1075	7.9	140	189	102	152
Coal Ash-Bottom Ash **^e^**	7.2	<5.5	191	20	0.018	na	73	262	123	59
Soil **^e^**	5.8	0.2	50	15	0.05	7	20	300	15	50
All Wood ash—all ash fractions **^f^**	13 (89)	6.5 (109)	57.2 (128)	59 (127)	0.4 (87)	9.1 (123)	97.7 (128)	7350 (122)	30 (127)	1595 (128)
Clean wood bottom ash **^f^**	<3 (32)	<0.51 (31)	49 (37)	15.5 (36)	<0.045 (28)	7.3 (37)	59 (37)	4900 (36)	20.5 (36)	400 (37)
Clean wood fly ash **^f^**	9.1 (26)	17 (30)	54 (31)	75 (31)	0.3 (28)	10 (26)	120 (31)	10850 (26)	31 (31)	3310 (31)

**Notes: ^a^** [171]; **^b^** [217]; **^c^** [170] **^d^** [9]; **^e^** [218] ; **^f^** [10].

**Table 12:** Other components, including persistent organics, measured in biomass ash.

Ash Fraction	C_org._ (wt% (d.b.))	Cl (wt% (d.b.))	PCDD/F (ng TE/kg d.b.)	PAH (mg/kg d.b.)	B[a]P (µg/kg d.b.)
Bark combustion					
Bottom ash	0.2–0.9	<0.06	0.3–11.7	1.4–1.8	1.4–39.7
Cyclone fly-ash	0.4–1.1	0.1–0.4	2.2–12.0	2.0–5.9	4.7–8.4
Filter fly-ash	0.6–4.6	0.6–6.0	7.7–12.7	137.0–195.0	900.0–4900.0
Wood chips combustion					
Bottom ash	0.2–1.9	<0.01	2.4–33.5	1.3–1.7	0.0–5.4
Cyclone fly-ash	0.3–3.1	0.1–0.5	16.3–23.3	27.6–61.0	188.0–880.0
Filter fly-ash	−	−	−	−	−
Pulverized Wood **^a^** Fly Ash				156	1500
Sawdust combustion					
Bottom ash	0.2–3.4	<0.1	1.3–2.1	14.7–21.1	21.0–40.5
Cyclone fly-ash	3.2–15.3	0.1–0.6	1.5–3.7	11.2–150.9	180.0–670.0
Filter fly-ash	−	−	−	−	−
Straw combustion					
Bottom ash	9.0	1.1	2.3	0.1	0.0
Cyclone fly-ash	16.6	13.6	70.8	15.8	17.0
Filter fly-ash	16.1	35.1	353.0	26.0	320.0
Cereal combustion					
Bottom ash	9.4	1.3	22.0	0.3	0.0
Cyclone fly-ash	9.9	5.2	12.2	0.5	0.0
Filter fly-ash	4.9	19.0	56.0	7.3	210.0

**Notes: ^a^** data from [175]; all other data from [9]; B[a]P = benzo[a]pyrene; C_org_ = Organic carbon; Cl = chlorine; d.b.= dry ash basis; PCDD/F = polychlorinated dibenzodioxin/furan; TE = Toxic equivalents standardized to toxicity of 2,3,7,8- tetrachlorodibenzo-*ρ*-dioxin (TCDD).

#### 4.3.4. Respirable Silica

Most biomass materials contain silica among the ash-forming material; the extent to which this silica can cause health effects via inhalation depends on the particle form and the fraction of the material that is respirable. Respirable free crystalline silica (*i.e.*, quartz) is associated with silicosis (a nodular pulmonary fibrosis), lung cancer, pulmonary tuberculosis, and other airway disorders [219]. Elevated risks are associated with occupations exposed to dust from rocks, including any activity involving sand blasting, brick cutting, rock drilling or blasting, *etc.* [219]. Exposure to coal ash results in exposure to respirable free silica, but no well-designed epidemiological study has established an association between silica exposure from this source and adverse health effects [162]. Some research has demonstrated that the lack of health effects may be because the free quartz in combusted material is vitrified and unable to interact with biological targets [163]. The tendency for silica in biomass ash to fuse has also been observed [9]. This feature, in conjunction with the understanding that in general biomass has a lower silica content than conventional solid fuel, indicates that the silica in ash is unlikely to pose an occupational health concern. In addition, some fluidized bed boilers use sand as a bed material; this material is removed with the ash (primarily the bottom ash) when degraded; levels of respirable quartz in this material are not clear. A study presented at the 2011 World of Coal Ash conference found that ash produced during the co-firing of biomass and coal had low levels of respirable quartz, which were not biologically available [163]. While the low silica content of biomass ash may be a general feature, an exception may be rice husks, which have particularly high quartz content [9]. From this perspective, the silica risk from the utilization of risk husks may be considered to be a potential concern for occupational exposures, but it is unlikely that other biomass ash would be a significant occupational concern. Still, more research to characterize the respirable quartz fraction of different biomass ash (and fuels) is warranted.

#### 4.3.5. Radioactivity

Concerns over the potential radioactivity of biomass ash stems from the expectation that natural or manufactured radioactivity present in plant material can become concentrated in ash upon combustion. Overall the concern has been less for natural radiation (which is generally considered to be negligible), and more for anthropogenic radionuclides that may be present at higher levels in plants and soils in areas that have experienced nuclear fall-out [167]. Principal radionuclides of concern are cesium-137, with a half-life (time taken for radioactivity to decay to 50% of the original levels) of 30.17 years and strontium-90 (half-life 28.8 years); the half-lives of these isotopes result in contamination remaining for many decades after the original event, and significant quantities of both were released from the Chernobyl and Fukushima accidents [220]. A limited number of studies have examined potential occupational radiation risk from biomass fuel. After an exposure assessment that included on-site monitoring of airborne dust, aerial radon, and ambient gamma dose-rate measurements, a study conducted at a peat-fired power station in Ireland concluded that workers involved in various plant activities did not experience a radiation dose above the level of concern established by the Irish government (calculated dose of 0.3 mSv per year against an action level of 1 mSv per year) [221]. Potential radiation exposures to workers have also been investigated in areas where the fuel stock is contaminated with radioactivity associated with the fall-out from Chernobyl.

## 5. Field Testing at Two Power Stations

### 5.1. Experience with Biomass Handling at UK Power Plant

Co-firing of biomass in coal-fired power plants started in the UK in around 2003 following the introduction of government requirements for renewable generation. Although co-firing ratios in most cases have been relatively low (<5% of thermal input), since most UK coal stations consist of 2 to 6 units rated between 330 and 660 MWe (with 4 × 500 MWe being the most common configuration), even at these low rates the quantities of biomass involved are significant. This has given these stations some experience with large-scale handling of biomass, with the storage and handling systems often subject to improvement from the initial design as experience increased. The biomass used in these projects has mainly been derived from agricultural residues such as palm kernel expeller, straw and olive cake, although energy crops including willow and miscanthus have also been used. In more recent years, regulatory support for biomass has moved towards high percentage (>50%) co-firing or dedicated biomass plants, through either “small”-scale new build stations (<50 MWe) using local biomass (often waste wood) or conversion of existing coal units to use biomass, with wood pellets the principal fuel used in these. These conversion projects, and similar conversions in continental Europe, represent the largest use of biomass in power generation globally. Most notably, Drax Power Station has recently completed conversion of two of its six 660 MWe units to 100% biomass firing, and is in the process of converting a further unit. When this project is completed, an estimated seven million tons of wood pellets will be required annually; this is compared to total global wood pellet production of 14 million tons per year in 2010 [222]. As a result of this and similar projects, the occupational health aspects of using biomass are becoming increasingly important, both at the power stations themselves as well as further upstream in the pellet production plants, ports and transport chains.

### 5.2. Testing and Analysis of Power Station Exposures

#### 5.2.1. Site Descriptions

In support of this review, testing of dust, fungal, and bacterial levels within two power stations was undertaken. Plant A is a 44MWe dedicated biomass CHP plant firing a mixture of fresh forestry chip (predominantly Northern European pine and spruce species), sawmill residues (derived from the same sources as the forestry chip), and reclaimed waste wood. The waste wood portion is source separated, so although the fuel includes particleboard as well as laminated, varnished, and painted material, wood treated with heavy metal-containing preservatives and chlorinated pesticides is specifically excluded. The boiler is a bubbling fluidized bed combustor with flue gas clean-up via activated carbon and lime injection into a bag filter (required due to EU regulations around the use of waste wood). The plant was visited on two occasions, in autumn and spring.

**Figure 1:**
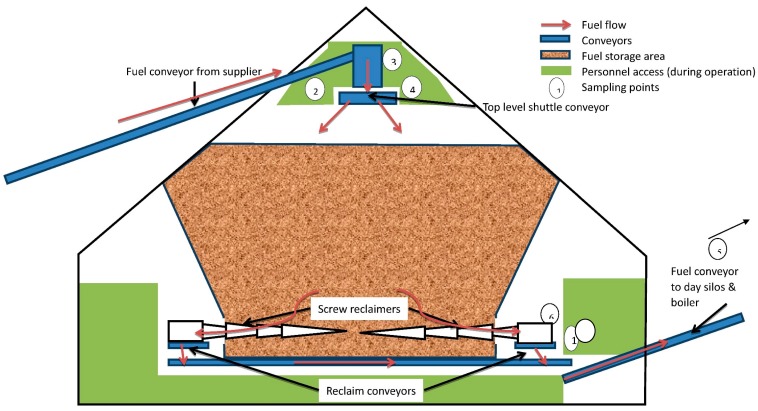
Schematic of fuel store layout at plant A (not to scale).

The fuel handling system at Plant A is relatively simple. The fuel is stored within a single “A-Frame” building capable of holding up to 5 days’ worth of fuel. Fuel is fed into the store by a central conveyor from the adjacent fuel supplier, which discharges at the roof-level of the store onto a shuttle conveyor, running approximately east-west, which distributes the fuel evenly across the stockpile below. Reclaim of the stock is via two screw reclaimers, each running along one side of the store, which discharge onto a common conveyor up to two “day silos” (each holding ~30 min of fuel) which feed the boiler. During plant operation there is no access along the conveyors from the store to the day silo, so sampling focused within the store, although at the autumn visit samples were also taken in the boiler house (Figure 1).

**Figure 2:**
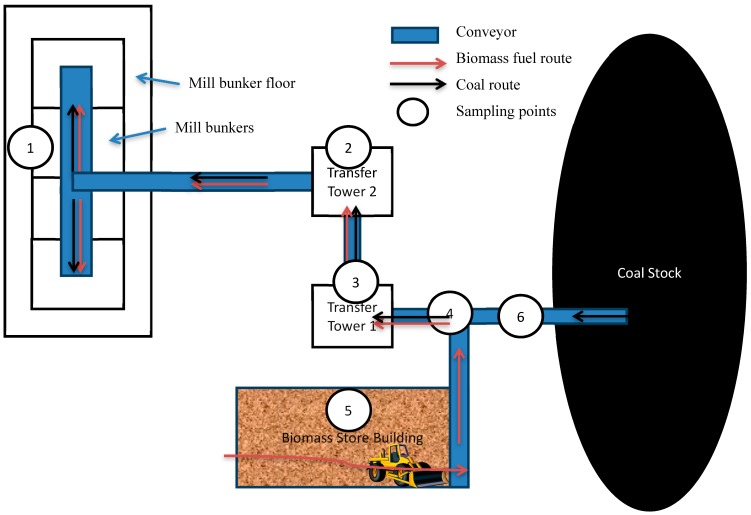
Schematic of handling system layout at plant B.

Plant B is a 2 GWe coal fired power station that has been co-firing various biomass types at levels of up to 15% for approximately 10 years. The plant consists of four pulverized coal units, all of which use cold-side electrostatic precipitators for dust control, while two units also have wet limestone flue gas desulfurization. The biomass is stored separately to the coal and added to the fuel by dosing the coal conveyors en route to the coal mills. At the time of testing, the biomass being co-fired was olive residue, at a percentage of around 3% thermal input. Access is available alongside all the conveyors, so testing was undertaken in the biomass store and at points in the conveyor system before the addition of biomass, where the biomass is added, in the two transfer towers and on the bunker floor of the mill house (Figure 2).

#### 5.2.2. Site Testing

At both plants, monitoring of inhalable dust levels was undertaken using a mixture of gravimetric personal exposure monitors and continuous dust monitors using laser scattering. At Plant B and during the second visit to Plant A, one continuous monitor was set up in a static location, identified by plant personnel as being both a common working area and prone to dust, while a second continuous monitor accompanied the test team. At various points in the conveyance system at each plant, identified in Figure 1 for Plant A and Figure 2 for Plant B, a Sartorius Airport MD8 instrument collected air samples onto gelatin filters for analysis for airborne microorganisms. Duplicate samples were taken at each location to enable spore identification and quantification of colony forming units. Sample volumes were 100 L at Plant A and 250 L at Plant B.

#### 5.2.3. Spore Quantification and Identification

Analyses for bacteria and fungi were undertaken as follows: one of each pair of sample filters was dispersed in 100 mL sterile Maximum Recovery Diluent. When fully dispersed, 0.5 mL of varying dilutions in MRD were each spread plated onto Nutrient Agar and Rose Bengal Chloramphenicol Agar with incubation at 30 °C for 2 days and 25 °C for 5 days respectively. Numbers of colony forming units were counted after these incubation periods and converted into equivalent levels in air using the known sample volumes.

Speciation of fungal spores was carried out by the National Pollen and Aerobiology Research Unit (NPARU) at the University of Worcester, UK. Filters were incubated on Malt Extract Agar to stimulate growth and generation of spores prior to microscopic (×400) identification of spores. Only those spores of health significance were considered and no quantification was undertaken.

### 5.3. Results and Discussion

#### 5.3.1. Levels of Bacteria and Fungi

**Figure 3:**
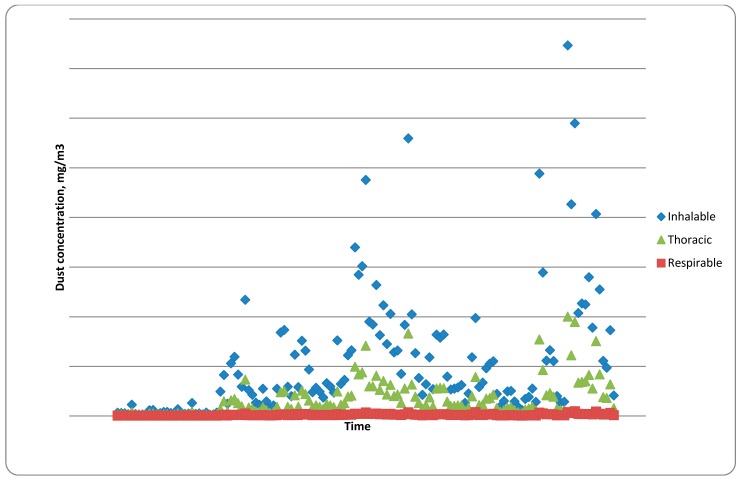
Measured dust levels adjacent to biomass feed hopper in plant B store shed.

**Figure 4:**
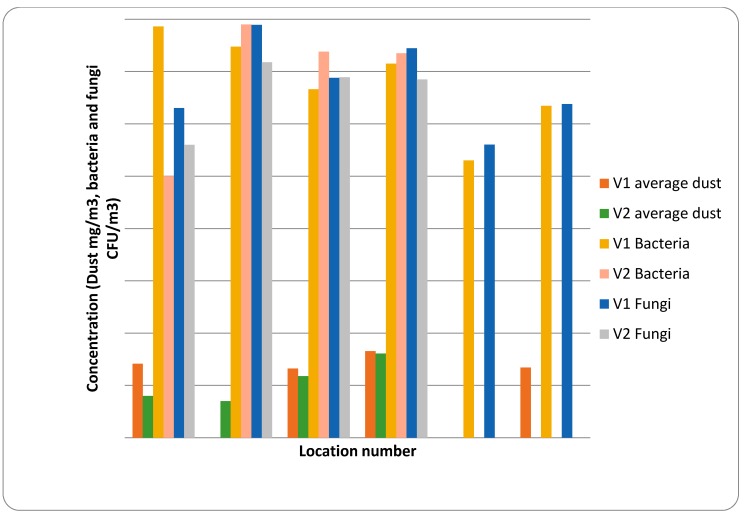
Dust, bacteria and fungi levels at plant A during visit 1 and visit 2 (note logarithmic scale). Limit of detection for bacteria and fungi is 3.98 × 10^2^.

The quantification of the number of colony forming units identified for both bacteria and fungi are shown in Table 13 and Table 14 and Figure 3 for Plant A, and Table 15 and Figure 4 for Plant B. Levels of bacteria peaked at 7.94 × 10^5^ cfu/m^3^ at Plant A and 1.51 × 10^4^ cfu/m^3^ at Plant B. As a point of comparison, Swan *et al.* (2003) reviewed a number of studies of bacteria in outdoor air and found average values of 79–3204 cfu/m^3^, with levels dependent on factors such as location and season. Peak levels of fungi were 7.8 × 10^5^ cfu/m^3^ for Plant A and 9.33 × 10^3^ cfu/m^3^ for Plant B. In outdoor air, Swan *et al.* (2003) reported highly variable fungal levels, ranging from close to zero to 9.4 × 10^4^ cfu/m^3^. These results suggest that levels of bacteria and fungi measured at these power plants were at the high end of what could be expected for an outdoor environment, with some results considerably higher and within the range where health effects have previously been observed. In comparison to other studies, the results from Plant B are similar to those observed in a Polish coal plant co-firing biomass [89].

#### 5.3.2. Types of Bacteria and Fungi

Bacterial types identified by the laboratory were not deemed to be of health significance and so were not reported. Included among these bacterial types were *Bacillus* species, but these were considered to be environmental species rather than either of the two *Bacillus* species of concern to health, *B. anthracis* and *B. cereus* (*B. anthracis* is not associated with plant biomass and a negative test for β-hemolytic activity when grown on Blood Agar excludes *B. cereus*).

Fungal types with potential health significance were also identified for each location, although exact species identification was not available. An overview of the fungal types, along with a summary of their health significance (provided by the testing laboratory) is provided in Table 16.

As seen in Table 20, some fungal types were more prevalent than others. *Penicillium* species were ubiquitous in both plants, appearing in all samples, but while *Paecilomyces* spp. were found at most locations in Plant A, only one location in Plant B yielded this species. *Mucor* spp. were found at fice of the six locations in Plant B but in only one sample from Plant A.

All of the fungal types identified are commonly found in the environment or associated with plant material. However, they include some of the fungal types most associated with health problems when handling biomass, in particular *Aspergillus* and *Penicillium* spp., which have previously been associated with allergic responses. The presence of these species highlights the need for adequate control measures to limit personnel exposure to fungal spores, and also the importance of health surveillance to identify those persons who may be more predisposed to health effects from exposure.

**Table 13:** Levels of Bacteria and Fungi at Plant A—Visit 1.

Sample	Colony Forming Units /m^3^		Genera of Health Significant Fungi Identified
	Bacteria	Fungi	
1.Screw reclaimer discharge onto conveyor to day silo	7.3 × 10^5^	2.0 × 10^5^	Mucor spp. Paecilomyces spp. Penicillium spp. Aspergillus spp. Yeast
2. Adjacent to shuttle conveyor, south side	3.0 × 10^5^	7.8 × 10^5^	Paecilomyces spp. Penicillium spp. Aspergillus spp.
3. Adjacent to fuel input conveyor	4.6 × 10^4^	7.6 × 10^4^	Paecilomyces spp. Penicillium spp. Aspergillus spp. Yeast
4. Adjacent to shuttle conveyor, north side	1.42 × 10^5^	2.8 × 10^5^	Paecilomyces spp. Penicillium spp. Yeast
Boiler house	<2.0 × 10^3^	4.0 × 10^3^	Paecilomyces spp. Penicillium spp. Mycelia sterilia
Adjacent to north side screw reclaimer	2.2 × 10^4^	2.4 × 10^4^	Paecilomyces spp. Penicillium spp. Aspergillus spp. Yeast

**Table 14:** Levels of Bacteria and Fungi at Plant A—Visit 2.

Sample	Colony Forming Units/m^3^		Genera of Health Significant Fungi Identified
	Bacteria	Fungi	
1.Screw reclaimer discharge onto conveyor to day silo	<1.00 × 10^3^	3.98 × 10^3^	*Paecilomyces* spp. *Penicillium* spp. Yeast
2. Adjacent to shuttle conveyor, south side	7.94 × 10^5^	1.51 × 10^5^	*Mucor* spp. *Paecilomyces* spp. *Penicillium* spp
3. Adjacent to fuel input conveyor	2.40 × 10^5^	7.76 × 10^4^	*Mucor* spp. *Penicillium* spp.
4. Adjacent to shuttle conveyor, north side	2.24 × 10^5^	7.08 × 10^4^	*Aspergillus* spp. *Mucor* spp. *Paecilomyces* spp. *Penicillium* spp.

**Table 15:** Levels of bacteria and fungi at plant B.

Sample	Colony Forming Units/m^3^		Genera of Health Significant Fungi Identified
	Bacteria	Fungi	
1. Mill bunker floor	1.51 × 10^4^	9.33 × 10^3^	*Aspergillus* spp. *Cladosporium* spp. *Mucor* spp. *Penicillium* spp.
2. Transfer tower 2	<3.98 × 10^2^	<3.98 × 10^2^	*Aspergillus* spp. *Mucor* spp. *Mycelia sterilia Penicillium* spp.
3. Transfer tower 1	<3.98 × 10^2^	2.82 × 10^3^	*Mucor* spp. *Paecilomyces* spp. *Penicillium* spp. Yeast
4. Biomass addition to coal conveyor point	1.20 × 10^3^	4.79 × 10^3^	*Mucor* spp. *Penicillium* spp.
5. Biomass store	<3.98 × 10^2^	<3.98 × 10^2^	*Mucor* spp. *Penicillium* spp.
6. Coal conveyor prior to biomass addition	<3.98 × 10^2^	7.41 × 10^3^	*Aspergillus* spp. *Penicillium* spp.

#### 5.3.3. Dust Levels in Plants

Summaries of the results from personal and static monitors at each plant are shown in Table 17, Table 18 and Table 19 and Figure 3 and Figure 4. Continuous monitoring results represent the exposures of the test team and so in some cases are averaged over relatively short periods (~15 min). Static monitoring results represent longer periods (generally 2–3 h). Longer-term (overnight) testing was attempted during the first visit to Plant A, but this was unsuccessful and the data are not presented.

Monitored dust levels in Plant A were surprising low, given visible evidence of dust accumulation on surfaces in the upper area of the store (sampling points 2–4). In the lower area of the store there was very little dust accumulation on surfaces. This reflects the different systems used in the store; in the upper level, the fuel free-falls from the input conveyor to the shuttle conveyor (although this is partially enclosed) and from there to the surface of the wood chip pile, creating opportunities for dust within the fuel to become airborne. In contrast, during the reclaim of the fuel, the screw augers are removing material from an essentially static pile and transfer distances between conveyors are much smaller, with the result that there is limited formation of dust. Discussions with the plant personnel indicate that the highest dust levels in the upper store are often seen during start-up of the conveyor system (due to the disturbance of settled dust) and when dry waste wood is being fed to the store, with the dust coming from degraded particle board. As the system was in continuous operation and primarily carrying wet forestry woodchip during the testing, it is probable that dust levels in the store are often significantly higher than those recorded. Under normal operation, plant personnel only spend limited time in the A-frame, primarily for cleaning and for maintenance checks; monitoring of the store is via video feed to the main control room. During start-up, access to the upper store is restricted; for general access to the upper level at other times, dust masks with a P3 rating (according to European standard EN149) are required as standard. However, when dust levels are visibly high, if undertaking work likely to generate airborne dust (such as cleaning), or if working for extended periods, air-fed hoods are used.

**Table 16:** Potential health implications of identified fungal types.

Fungal Group	Health Significance
*Aspergillus* spp.	Common environmental organism being found in soil, plant debris, decaying fruit and vegetables as well as indoor environments. Can act as a potent allergen causing allergic asthma with some species producing mycotoxins. Some species can cause infection in humans invading the lungs, sinuses and other sites sometimes causing deep infections in immunocompromised persons. Non-immunocompromised persons may also occasionally show infection of sinuses and lungs.
*Mucor* spp.	Widespread in soil, plants, decaying vegetation *etc.* May cause zygomycosis or mucormycosis in humans—infection of nose, septic arthritis, dialysis-associated peritonitis, renal infections, gastritis and lung infections. Exacerbated by persons being immunocompromised or being diabetic
*Penicillium* spp.	Widespread throughout environment especially associated with soil and decaying vegetation. May cause allergic asthma and lead to irritation of respiratory tract. May occasionally cause more serious illness with species capable of producing mycotoxin.
*Paecilomyces* spp.	An inhabitant of soil and decaying vegetation, occasionally found in foods and in air. Often isolated from compost. May give rise to allergic reactions with the immunocompromised most at risk.
Yeasts	Common airborne fungus. May be a problem if a person has been previously exposed and has become hypersensitive. High levels may cause allergies.
*Mycelia sterilia*	Ubiquitous with some being important plant pathogens.
*Cladosporium* spp.	Widely distributed in air and rotten organic material and is frequently isolated from foods. Infection may lead to skin lesions, keratitis, nail infections, sinusitis and lung infection.

**Table 17:** Average and maximum inhalable dust levels at plant A—visit 1.

Location number	Continuous Monitor	
	Average inhalable dust level, mg/m^3^	Maximum inhalable dust level, mg/m^3^
1	0.26	0.39
3	0.21	0.32
4	0.45	1.30
6	0.22	0.67

**Figure 5:**
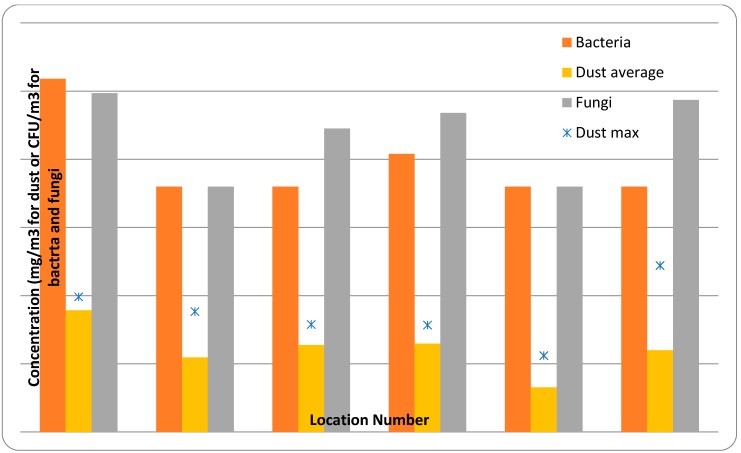
Dust, bacteria and fungi levels at plant B (note logarithmic scale). Limit of detection for bacteria and fungi is 3.98 × 10^2^.

Levels of dust at Plant B were generally higher than at Plant A. Within the storage shed at Plant B, vehicle movements restricted access of the test team, so the continuous dust monitor results presented are for an area away from the main working zone. It was, however, possible to set up a continuous monitor as a static monitor closer to the area where the biomass is fed onto the conveyance system using a front loader. The results from this monitor are shown in Figure 5. It can be seen that ambient dust levels in the storage shed were low prior to the start of operations at 10:30, but after this time there were occasional high levels (up to 37 mg/m^3^) of inhalable dust—most likely representing tipping operations. Levels of respirable dust remained low (<1 mg/m^3^) throughout, indicating that the dust generated was inhalable but not respirable. This is the only area at the plant where it can be assumed that the majority of the dust exposure is from the biomass itself (there may also be a contribution from the diesel vehicles), as throughout the rest of the plant there is a contribution from coal. This can be seen with the results from location 6, where only coal dust is expected, but the second highest maximum dust level was seen. The results of the gravimetric personnel monitor accompanying the test team were nearly 10× higher than those from the continuous monitor (14 mg/m^3^ verses 1.84 mg/m^3^) and above the UK workplace exposure limit for inhalable dust of 10 mg/m^3^. It is not clear whether this difference in results is “real” and reflects the variability of monitoring in an area of changing conditions, or is indicative of limitations in sampling methods. For example, disturbance to dust accumulated on surfaces while moving around the plant may create localized areas of very high dust concentrations that may be picked up by one monitor but not the other. In addition, non-inhalable dust may settle onto gravimetric filters, artificially increasing the collected mass, or the characteristics of the dust may make it difficult for continuous systems to detect.

**Table 18:** Average and maximum inhalable dust levels at plant A—visit 2.

Location number	Continuous Monitor		Static Monitors
	Average inhalable dust level, mg/m^3^	Maximum inhalable dust level, mg/m^3^	Average inhalable dust level, mg/m^3^
1	0.063	3.4	
2	0.05	1.83	1.10
3 (static monitor)	0.37	2.46	0.55
3 (test team monitor)	0.15	1.59	
4	0.405	1.65	
Outside	0.21	6.8	

**Table 19:** Average and maximum inhalable dust levels at plant B.

Location number	Continuous Monitor		Gravimetric Monitor
	Average inhalable dust level, mg/m^3^	Maximum inhalable dust level, mg/m^3^	Average inhalable dust level, mg/m^3^
1	6.10	9.64	
2	1.24	5.85	
3	1.89	3.78	
4	1.98	3.71	
5 (static monitor)	5.31	37.34	4.00
5 (test team monitor)	0.45	1.32	
6	1.58	27.66	
Coal plant control room	0.25	0.57	
Outside	0.41	2.11	
Test team gravimetric monitor			14.23

**Table 20:** Summary of fungal species identified.

Site	Plant A										Plant B					
	1. Screw reclaimer discharge onto conveyor to day silo		2. Adjacent to shuttle conveyor, south side		3. Adjacent to fuel input conveyor		4. Adjacent to shuttle conveyor, north side		5. Boiler house	6. Adjacent to north side screw reclaimer	1. Mill bunker floor	2. Transfer tower 2	3. Transfer tower 1	4. Biomass addition to coal conveyor point	5. Biomass store	6. Coal conveyor prior to biomass addition
Visit	1	2	1	2	1	2	1	2	1	1						
Identified fungal types																
*Mucor* spp.	√			√		√		√			√	√	√	√	√	
*Paecilomyces* spp.	√	√	√	√	√		√	√	√	√			√			
*Penicillium* spp.	√	√	√	√	√	√	√	√	√	√	√	√	√	√	√	√
*Aspergillus* spp.	√		√		√			√		√	√	√				√
Yeast	√	√			√		√			√			√			
*Mycelia sterilia*									√			√				
*Cladosporium* spp.											√					

### 5.4. Conclusions of Field Sampling

This limited study highlights some of the additional hazards associated with the use of biomass for power generation compared with coal generation. The exposure levels to dust, fungi, and bacteria varied between the two plants monitored, being affected by factors such as the location within the plant, activities in the immediate area of the monitors, and most likely other factors, including the biomass type and seasonal conditions. The discrepancy between gravimetric and continuous dust monitors located in the same area of the plant highlights the uncertainties associated with any monitoring campaign and the influencing factors that must be considered. Levels of bacteria and fungi were within the range seen in other related industries such as agriculture and composting, but were generally higher than levels considered typical of ambient outdoor air. They were also above the levels where health effects have been identified in previous studies. The types of fungi identified were all commonly found in the environment, but included some such as *Aspergillus* and *Penicillium* species which are known to be associated with allergic and respiratory effects. It is therefore important that the risk to workers is properly evaluated when biomass is considered as a fuel, with factors such as biomass type, handling, and storage methods and the interaction between workers and biomass taken into consideration. Where possible, good practice should be used to minimize the risk of bioaerosol formation; methods could include minimization of storage times and avoidance of conditions which could promote mold growth (such as accumulation of biomass dust in warm, moist conditions). Process control of occupational exposure risks is preferable to reliance on personal protective equipment due to factors such as proper fit and maintenance which can reduce the protection factor of, e.g., dust masks.

## 6. Conclusions

The aim of this review was to summarize the state of knowledge regarding potential occupational hazards related to biomass-powered electricity generation. Due to the limited number of publically available occupational monitoring, assessment, or epidemiological studies, it provides an overview based primarily on extrapolation of potential exposures and adverse health outcomes derived from diverse industrial hygiene, laboratory and epidemiological work conducted in related wood or agricultural industries, or other ambient exposure scenarios, including uncontrolled biomass burning, in non-worker populations.

However, even with this severe limitation, this qualitative extrapolation does provide indications of potential hazards associated with the use of biomass that are not regularly encountered in fossil-based power generation, which should be considered in the context of protecting worker health through the development of monitoring and control plans. Pre-combustion risks include the following: particulate matter containing bioaerosols and biogenic organics such as fungi, bacteria, and other microbial components capable of inducing irritation (e.g., ocular and dermal), acute or chronic allergic responses (e.g., dermatitis, rhinitis, or conjunctivitis) and chronic allergic responses (e.g., occupational asthma). Additionally, as IARC classifies at least some wood dust as carcinogenic, it remains prudent to control dust levels, particularly as for many authorities lower OELs are specified for wood dust than for general dust. As an organic fuel, biomass lacks the stability of traditional coal or petroleum fuels and has a tendency to decompose, create changing exposure scenarios and requiring different handling, transport, and storage considerations to minimize both microbial growth (e.g., spore formation, endotoxin release, *etc.*) and off-gassing of volatile organics or other gases (e.g., carbon monoxide). Where this degradation cannot be avoided, specific monitoring and control programs may be required. It remains to be seen if biomass applications in the power sector put workers at higher risk of more severe respiratory diseases observed in agriculture or other industries, such as organic dust toxic syndrome or allergic alveolitis (e.g., Farmers Lung). Regardless, proactive training on unique handling practices and health surveillance focused on respiratory considerations for workers will not only provide a safety buffer, but also encourage and provide data to support monitoring, occupational exposure and risk assessments.

Combustion and post-combustion occupational exposures, along with related health and safety concerns, appear likely to mirror current, more traditional combustion scenarios. In addition to appropriate technology controls, worker training on appropriate ash handling during operational and maintenance procedures will parallel current best practices. However, the available data on biomass physiochemical properties as they relate to emissions and solid waste streams indicate that some of the hazards may be different to those from fossil-based generation, particularly when using waste fuels, and so this should be considered when evaluating worker risk.

Limited public domain information is available from on-going health and injury surveillance of power generation workers, particularly for health outcomes of highest concern (e.g., respiratory, irritation, sensitization). Additional studies at power plants utilizing a variety of technologies and biomass stock fuels, particularly with personal and task specific monitoring, may be required to understand the background prevalence of symptoms and disease among workers and move health and safety research forward as the global interest in, and application of, biomass as a renewable energy source increases.
